# Supplementary material for: Polymicrobial bloodstream infections a risk factor for mortality in neonates at the national hospital, Tanzania: A case-control study
Source: PLoS One. 2024 Apr 16;19(4):e0302076. doi: 10.1371/journal.pone.0302076 (PMC11020784; doi:10.1371/journal.pone.0302076)
Supplement: Attachment — Submitted filename: PONE-D-23-28734_R1.pdf [file pone.0302076.s004.pdf]

PLOS ONE

Polymicrobial bloodstream infections a risk factor for mortality in neonates at the national hospital, Tanzania: A case-control study

--Manuscript Draft--

**Manuscript Number:**	PONE-D-23-28734R1
**Article Type:**	Research Article
**Full Title:**	Polymicrobial bloodstream infections a risk factor for mortality in neonates at the national hospital, Tanzania: A case-control study
**Short Title:**	Polymicrobial, bloodstream infections, mortality, Tanzania
**Corresponding Author:**	Joel Manyahi<br>Muhimbili University of Health and Allied Sciences School of Medicine<br>Dar es Salaam, TANZANIA, UNITED REPUBLIC OF
**Keywords:**	Polymicrobial, bloodstream infections, mortality, Tanzania
**Abstract:**	Background: Polymicrobial bloodstream infections (BSI) are difficult to treat since empiric antibiotics are frequently less effective against multiple pathogens. The study aimed to compare outcomes in patients with polymicrobial and monomicrobial BSIs.<br>Methods: The study was a retrospective case-control design conducted at Muhimbili National Hospital for data processed between July 2021 and June 2022. Cases were patients with polymicrobial BSI, and controls had monomicrobial BSI. Each case was matched to three controls by age, admitting ward, and duration of admission. Logistic regression was performed to determine independent risk factors for in-hospital and 30-day mortality.<br>Results: Fifty patients with polymicrobial BSI and 150 with monomicrobial BSI were compared: the two had no significant differences in sex and comorbidities. The most frequent bacteria in polymicrobial BSI were Klebsiella pneumoniae 17% (17/100) and Enterobacter species 15% (15/100). In monomicrobial BSI, S. aureus 17.33% (26/150),Klebsiella pneumoniae 16.67% (25/150), and Acinetobacter species 15% (15/150) were more prevalent. Overall, isolates were frequently resistant to multiple antibiotics tested, and 52% (130/250) were multidrug resistance. The 30-day and in-hospital mortality were 33.5% (67/200) and 36% (72/200), respectively. On multivariable analysis, polymicrobial BSIs were independent risk factors for both in-hospital mortality (aOR 2.37, 95%CI 1.20-4.69, p = 0.01) and 30-day mortality (aOR 2.05, 95%CI 1.03-4.08), p = 0.04). In sub-analyses involving only neonates, polymicrobial BSI was an independent risk factor for both 30-day mortality (aOR 3.13, 95%CI 1.07-9.10, p = 0.04) and in-hospital mortality (aOR 5.08, 95%CI 1.60-16.14, p = 0.006). Overall, the median length of hospital stay post-BSIs was numerically longer in patients with polymicrobial BSIs.<br>Conclusion: Overall, mortality was significantly frequent in patients with polymicrobial BSI. Patients with polymicrobial BSI stay long at the hospital than monomicrobial BSI. These findings call for clinicians to be more aggressive in managing polymicrobial BSI.
**Order of Authors:**	Joel Manyahi, PhD
	Agricola Joachim, PhD
	Frank Msafiri, PhD
	Mary Migiro, BMLS
	Anthon Mwingwa, MMed
	Mabula Kasubi
	Helga Naburi
	Mtebe Majigo
**Response to Reviewers:**	Reviewer #1: The evidence for polymicrobial BSI is not very clear just isolation of two or more bacteria from blood culture does not substantiate the polymicrobial BSI.

Powered by Editorial Manager® and ProduXion Manager® from Aries Systems Corporation

Comment: Line 164 and 165 Acinetobacter and CONS, Pseudomonas and S.aureus in these combinations CONS and S.aureus could be skin contaminants in which case BSI is mono-microbial only. Therefore, selection criteria of polymicrobial BSI cases to be clearly defined in which clinical conditions or comorbidities polymicrobial BSI is a possibility only such case to be selected.
Response: Thank you for the comment. Our laboratory SoP for a blood culture states very clearly when to consider CoNS, and other possible skin contaminants as true pathogens, and we strictly adhered to the laboratory SoP in defining pathogens. S. aureus in blood culture is always considered a pathogen in our interpretation of the blood culture positive

Comment: Line 166 and 167 Acinetobacter 15% (15/150) calculation error.
Response: We appreciate for the comment, we have addressed calculation error

Comment: While mentioning the antibiotic resistance consider the antibiotics used for blood stream infections not clindamycin, cotrimoxazole and chloramphenicol for S. aureus.
Response: We appreciate for the comment. However, our results show the susceptibility pattern based on laboratory testing and not based treatment of S. aureus blood stream infections. In reporting AST results to clinician, our laboratory considers both bacteria and site of infections.

Comment: Which are the disease comorbidities were considered for statistical analysis because these disease comorbidities will play very significant role in hospital mortality and 30-day mortality in patients with BSI besides the antimicrobial resistance.
Response: Unfortunately, clinical conditions were so diverse, and a breakdown presentation was difficulties. Therefore, in our analysis we categorized either presence of comorbidity or not.

Reviewer #2: The paper made an interesting reading, dealing with polymicrobial infections Vs Monomicrobial Infections , the microbiological and clinical impact of these with respect to 30 day mortality and the extended hospital stay etc.
Comments for the same are attached , the authors are advised to kindly refer to the same and address all the points mentioned

The paper deals with the differences (Both Microbiological yield and clinical outcomes) in patients with Polymicrobial and Monomicrobial Bacteremia Infections; The Following observations are in order and must be addressed before the manuscript can be taken forward

Comment: The title of the paper does not reveal that the major component of the study is in children, adolescents, neonates, infants or adults. However, the Table No 4 & 5 refer to clinical outcomes in neonates only.
Response: Thank you for the comment. We agree that the title does not mention the major components of the participants. Our study aimed at creating awareness of the negative effects of polymicrobial bloodstreams on patient care for both laboratory personnel and clinicians. Being a lab-based retrospective, we therefore enrolled all patients with polymicrobial bloodstreams matching their counterparts by age, date of admission, and admitting wards. However, because we had a significant number of participants who were neonates, we decided to perform a sub-analysis for neonates only, as appears in Tables 4 and 5. On the other hand, we have done sub-analysis in none neonates, Therefore, we have modified the title to show the component affected.

Comment: Moreover, the Table – 1 which deals with age and sex distribution does not give a breakdown of the age groups and refers to a median age alone.
Response: Thank you for the comment. Our study design was a case control, which was matched by age; therefore, we found most participants were populated in the same age category. However, we have added age breakdown in table1

Comment: The discussion section refers to certain observations which have been compared with other studies and these deal with outcomes in neonates only.
Response: We appreciate for the comment, we have improved our citation and omitted references which do not match with our study population

Powered by Editorial Manager® and ProduXion Manager® from Aries Systems Corporation

Comment: In the section “Materials and Methods “ “study setting and design ‘ the authors have stated that results are obtained in age groups from 1-86 years and of these 50 patients were selected and 150 controls. What was the age distribution taken into consideration when this selection was made. The clinical outcomes may be different in different age groups with polymicrobial bacteremia.
Response: We appreciate for the good comment, in this study we included all age groups from 0-86. Understanding the age have influence on the clinical outcome, first we adjusted for age in multivariable analysis. Then we did stratification analysis for neonate only and non-neonate. Having this analysis, we are assured we have controlled the age a possible confounder influencing our outcome of interest.

Comment: The authors have stated a definition for Polymicrobial infections in the section “Definition of terms”. However, the term polymicrobial need not be restricted to 2 organisms as there are instances of blood stream infections caused by more than 2 organisms, including a yeast. How has the selection for polymicrobial infections data been made. Moreover the Results section talks of common organisms as Nos/ 100 which means that the authors have considered only 2 organisms per blood culture in a patient (total no of patients being 50).
Response: Thank you for this observation. We completely agree polymicrobial infections can be caused by two or more bacteria. Unfortunate, during the review of the laboratory data, we did not come to an instance of having more than two pathogens. Furthermore, we had one case of polymicrobial bloodstream infections involving bacteria and candida species.

Comment: The clinical outcomes for blood stream infections in conditions such as Perforation peritonitis, Carcinoma colon etc may depend on the nos of organisms causing the Polymicrobial Infection. Has this been taken into account. There is no mention anywhere in the manuscript and yeasts do not form part of the study.
Response: In our analysis we considered co-morbidities as one of the confounders, which could influence our outcome of interest, and in multivariable analysis we controlled for comorbidities, however, we found this did not influence our outcome. Furthermore, we did not document any case with perforation or peritonitis, but there was only one case of polymicrobial infections in patient with rectal carcinoma. In table 2, we have mentioned yeast being part of the study.

Comment: The authors have stated that Viridans streptococcus and Corynebacteria have been disregarded as contaminants. However, these organisms may assume huge clinical significance in certain clinical situations such as immunocompromise and malignancies in patients with BSI’s. The authors have not provided a break up of clinical conditions in the 50 cases and 150controls which in itself may skew the clinical outcome data of patients.
Response: Thank you for the comment. Our laboratory SoP for a blood culture states very clearly when to consider CoNS, Viridans Streptococcus, Corynebacterium as true pathogens, and we strictly followed the lab SoP regarding these as pathogenic. Unfortunately, clinical conditions were diverse, and a breakdown presentation in a table would be difficult for a reader.

Comment: The description of Results section under “Antimicrobial Susceptibility Pattern” does not match the data outlined in Table -3.
Response: Thank you for observation, we have edited on the total number of MDR bacteria

Comment: There is no reference to certain Resistance mechanisms such as Carbapenem resistant Enterobacterales (CRE), Amp C enzymes MLSBi/c detection in S. aureus. All of these do impact treatment outcomes. How have these been factored in the selection of 50 cases and 150 controls in the study as these would most certainly affect the final analyses
Response: Thank you for the valid comment. In the selection of cases and controls, we did not consider if patients had resistant strains or not because our study hypothesized that polymicrobial bloodstream infections were associated with poor treatment outcomes, and we designed this study as a case control to answer our research question. We had thought of controlling for MDR or resistant strains, but the analysis could have been different and not for a case-control study, maybe cross-sectional.

Powered by Editorial Manager® and ProduXion Manager® from Aries Systems Corporation

	Comment: The section on Discussion gives vague references to 30-day mortality and extended hospital stay etc without actual figures and how these may compare with similar data brought out in other studies. There are equal references to other studies in neonates without actually discussing the distribution of cases among neonates in the present study.<br>Response: Thank you for the comments, we have improved our reference to include reference from similar study group.<br><br>Comment: Based on the data presented and discussed the authors may have to modify the conclusion section as no definite conclusions emerge out of the discussion cited.<br>Response: Thank you, we have rephrased our conclusion.
**Additional Information:**	
**Question**	**Response**
**Financial Disclosure**<br><br>Enter a financial disclosure statement that describes the sources of funding for the work included in this submission. Review the submission guidelines for detailed requirements. View published research articles from *PLOS ONE* for specific examples.<br><br>This statement is required for submission and **will appear in the published article** if the submission is accepted. Please make sure it is accurate.<br><br>**Unfunded studies**<br>Enter: *The author(s) received no specific funding for this work.*<br><br>**Funded studies**<br>Enter a statement with the following details:<br>• Initials of the authors who received each award<br>• Grant numbers awarded to each author<br>• The full name of each funder<br>• URL of each funder website<br>• Did the sponsors or funders play any role in the study design, data collection and analysis, decision to publish, or preparation of the manuscript?<br>• **NO** - Include this sentence at the end of your statement: *The funders had no role in study design, data collection and analysis, decision to publish, or preparation of the manuscript.*<br>• **YES** - Specify the role(s) played.<br><br>* typeset	The author(s) received no specific funding for this work

**Competing Interests**<br><br>Use the instructions below to enter a competing interest statement for this submission. On behalf of all authors, disclose any competing interests that could be perceived to bias this work—acknowledging all financial support and any other relevant financial or non-financial competing interests.<br><br>This statement is required for submission and **will appear in the published article** if the submission is accepted. Please make sure it is accurate and that any funding sources listed in your Funding Information later in the submission form are also declared in your Financial Disclosure statement.<br><br>View published research articles from *PLOS ONE* for specific examples.<br><br>**NO authors have competing interests**<br><br>Enter: *The authors have declared that no competing interests exist.*<br><br>**Authors with competing interests**<br><br>Enter competing interest details beginning with this statement:<br><br>*I have read the journal's policy and the authors of this manuscript have the following competing interests: [insert competing interests here]*<br><br>* typeset	The authors have declared that no competing interests exist
**Ethics Statement**<br><br>Enter an ethics statement for this submission. This statement is required if the study involved:<br><br>• Human participants<br>• Human specimens or tissue<br>• Vertebrate animals or cephalopods	Ethical approval was granted by the Muhimbili University of Health and Allied Sciences senate and publications committee Ref.No DA.25/111/01C/157. Permission to conduct the study was approved by Muhimbili National Hospital management. As the study was a retrospective study using identified patients, informed consent was waived by Muhimbili University of Health and Allied Sciences senate and publication committee. All methods were carried out under relevant guidelines and regulations in the declaration.

• Vertebrate embryos or tissues<br>• Field research<br><br>Write "N/A" if the submission does not require an ethics statement.<br><br>General guidance is provided below. Consult the submission guidelines for detailed instructions. **Make sure that all information entered here is included in the Methods section of the manuscript.**	

Powered by Editorial Manager® and ProduXion Manager® from Aries Systems Corporation

**Format for specific study types**<br><br>**Human Subject Research (involving human participants and/or tissue)**<br>• Give the name of the institutional review board or ethics committee that approved the study<br>• Include the approval number and/or a statement indicating approval of this research<br>• Indicate the form of consent obtained (written/oral) or the reason that consent was not obtained (e.g. the data were analyzed anonymously)<br><br>**Animal Research (involving vertebrate animals, embryos or tissues)**<br>• Provide the name of the Institutional Animal Care and Use Committee (IACUC) or other relevant ethics board that reviewed the study protocol, and indicate whether they approved this research or granted a formal waiver of ethical approval<br>• Include an approval number if one was obtained<br>• If the study involved *non-human primates*, add *additional details* about animal welfare and steps taken to ameliorate suffering<br>• If anesthesia, euthanasia, or any kind of animal sacrifice is part of the study, include briefly which substances and/or methods were applied<br><br>**Field Research**<br><br>Include the following details if this study involves the collection of plant, animal, or other materials from a natural setting:<br>• Field permit number<br>• Name of the institution or relevant body that granted permission	
**Data Availability**<br><br>Authors are required to make all data underlying the findings described fully available, without restriction, and from the time of publication. PLOS allows rare exceptions to address legal and ethical concerns. See the PLOS Data Policy and FAQ for detailed information.	Yes - all data are fully available without restriction

A Data Availability Statement describing where the data can be found is required at submission. Your answers to this question constitute the Data Availability Statement and **will be published in the article**, if accepted.<br><br>**Important:** Stating 'data available on request from the author' is not sufficient. If your data are only available upon request, select 'No' for the first question and explain your exceptional situation in the text box.<br><br>Do the authors confirm that all data underlying the findings described in their manuscript are fully available without restriction?	
**Describe where the data may be found in full sentences. If you are copying our sample text, replace any instances of XXX with the appropriate details.**<br><br>• If the data are **held or will be held in a public repository**, include URLs, accession numbers or DOIs. If this information will only be available after acceptance, indicate this by ticking the box below. For example: *All XXX files are available from the XXX database (accession number(s) XXX, XXX.*).<br>• If the data are all contained **within the manuscript and/or Supporting Information files**, enter the following: *All relevant data are within the manuscript and its Supporting Information files.*<br>• If neither of these applies but you are able to provide **details of access elsewhere**, with or without limitations, please do so. For example:<br><br>*Data cannot be shared publicly because of [XXX]. Data are available from the XXX Institutional Data Access / Ethics Committee (contact via XXX) for researchers who meet the criteria for access to confidential data.*<br><br>*The data underlying the results presented in the study are available from (include the name of the third party*	Information will be only be available after acceptance

and contact information or URL).<br>• This text is appropriate if the data are owned by a third party and authors do not have permission to share the data.<br><br>* typeset	
Additional data availability information:	Tick here if the URLs/accession numbers/DOIs will be available only after acceptance of the manuscript for publication so that we can ensure their inclusion before publication.

Powered by Editorial Manager® and ProduXion Manager® from Aries Systems Corporation

Manuscript Click here to access/download;Manuscript;Manuscript_JM.docx

Polymicrobial bloodstream infections a risk factor for mortality in neonates at the national hospital, Tanzania: A case-control study

Authors: Joel Manyahi[1*], Agricola Joachim[1], Frank Msafiri[1], Mary Migiro[1], Anthon Mwingwa[1], Mabula Kasubi[2], Helga Naburi[3], Mtebe Venance Majigo[1]

Affiliations:

[1]Department of Microbiology and Immunology, Muhimbili University of Health and Allied Sciences, Dar es Salaam, Tanzania.

[2]Muhimbili National Hospital, Dar es Salaam, Tanzania.

[3]Department of Pediatrics and Child Health, Muhimbili University of Health and Allied Sciences, Dar es Salaam, Tanzania.

Email:

Joel Manyahi (manyahijoel@yahoo.com)

Agricola Joachim (agricolaj@yahoo.com)

Frank Msafiri (frankmbulinyingi@yahoo.com)

Mary Migiro (marymigiro03@gmail.com)

Anthon Mwingwa (anthonymwingwa@gmail.com)

Mabula Kasubi (Mkasubi68@gmail.com)

Helga Naburi (hnaburi2000@yahoo.com

Mtebe **Venance** Majigo (mmajigo@gmail.com)

Corresponding Author*

Joel Manyahi, Muhimbili University of Health and Allied Sciences, P.O. Box 65001, Dar es Salaam, Tanzania

Mobile Number +255712251709

Keywords: Polymicrobial, bloodstream infections, mortality, Tanzania

Abstract

Background: Polymicrobial bloodstream infections (BSI) are difficult to treat since empiric antibiotics are frequently less effective against multiple pathogens. The study aimed to compare outcomes in patients with polymicrobial and monomicrobial BSIs.

Methods: The study was a retrospective case-control design conducted at Muhimbili National Hospital for data processed between July 2021 and June 2022. Cases were patients with polymicrobial BSI, and controls had monomicrobial BSI. Each case was matched to three controls by age, admitting ward, and duration of admission. Logistic regression was performed to determine independent risk factors for in-hospital and 30-day mortality.

Results: Fifty patients with polymicrobial BSI and 150 with monomicrobial BSI were compared: the two had no significant differences in sex and comorbidities. The most frequent bacteria in polymicrobial BSI were *Klebsiella pneumoniae* 17% (17/100) and *Enterobacter* species 15% (15/100). In monomicrobial BSI, *S. aureus* 17.33% (26/150), *Klebsiella pneumoniae* 16.67% (25/150), and *Acinetobacter* species 15% (15/150) were more prevalent. Overall, isolates were frequently resistant to multiple antibiotics tested, and 52% (130/250) were multidrug resistance. The 30-day and in-hospital mortality were 33.5% (67/200) and 36% (72/200), respectively. On multivariable analysis, polymicrobial BSIs were independent risk factors for both in-hospital mortality (aOR 2.37, 95%CI 1.20-4.69, p = 0.01) and 30-day mortality (aOR 2.05, 95%CI 1.03-4.08), p = 0.04). In sub-analyses involving only neonates, polymicrobial BSI was an independent risk factor for both 30-day mortality (aOR 3.13, 95%CI 1.07-9.10, p = 0.04) and in-hospital mortality (aOR 5.08, 95%CI 1.60-16.14, p = 0.006). Overall, the median length of hospital stay post-BSIs was numerically longer in patients with polymicrobial BSIs.

Conclusion: Overall, mortality was significantly frequent in patients with polymicrobial BSI. Patients with polymicrobial BSI stay long at the hospital than monomicrobial BSI. These findings call for clinicians to be more aggressive in managing polymicrobial BSI.

Background

Bloodstream infections (BSI) in low-middle-income countries are steadily associated with poor treatment outcomes, with the rising concern associated with antibiotic-resistant bacteria. Bacteremia has been reported in 10–13% of inpatients with severe febrile illness in East and West Africa, with an estimated case fatality rate of 12% [1, 2].

Following the reduction of malaria, a frequent cause of febrile illness in Tanzania, BSIs remain a common cause of hospitalization in children with febrile illness. In Tanzania, BSIs is detected in 11–14% of children with severe febrile illness [3, 4]. Studies have shown that BSIs in adults and children result in worse outcomes, such as high mortality and length of hospital stay [5-8]. In addition, increasing evidence of antibiotic-resistant bacteria, including methicillin-resistant *Staphylococcus aureus* (MRSA), extended-spectrum beta-lactamases (ESBL), and multidrug-resistant (MDR) bacteria in BSIs, has been reported in Tanzania [3-5, 9].

Studies in Tanzania have reported that BSIs due to antibiotic-resistant bacteria had a significantly higher risk of death than those with antibiotic susceptible bacteria [5, 8, 9]. Mortality in children with ESBL septicemia was approximately 2-fold higher than in non-ESBL septicemia [9]. Our recent study found that patients with MDR BSIs (aOR = 15) had a higher risk of dying than those with non-MDR BSIs [5]. However, studies still need to evaluate the outcomes of polymicrobial BSIs in Tanzania thoroughly.

Polymicrobial BSIs are frequently difficult to treat, and choosing the appropriate empiric antibiotics effective for more than one pathogen is more challenging. It is, therefore, vital to

promptly identify the pathogens causing polymicrobial BSIs and administer the proper empiric antibiotics/drugs. In addition, studies on adults and children from other settings have demonstrated that mortality attributable to polymicrobial BSIs is higher than in monomicrobial BSIs [10-13]. Therefore, we carried out this retrospective case-control study at Muhimbili National Hospital (MNH) to ascertain the impact of polymicrobial BSIs on patient outcomes. A better understanding of the risk factors for worse outcomes in patients with BSIs would improve clinical outcomes for patients.

Materials and methods

Study setting and design

This was a retrospective case-control study for the data processed between July 2021 and June 2022 at MNH, Dar es Salaam, Tanzania. The data for this study were accessed and collected between July 1, 2022, and September 30, 2022. MNH is a tertiary-level hospital in Tanzania and serves as a teaching hospital for Muhimbili University of Health and Allied Sciences (MUHAS). It has a 1,500-bed capacity and attends up to 2,000 outpatients daily. The hospital has an infection control policy and an infection prevention and control (IPC) unit. Furthermore, blood culture sample in all wards is collected under aseptic condition and transferred to the laboratory within one hour of collection. Under ideal situations, the laboratory reporting system allows to flag clinicians with preliminary results immediately when the blood culture flag-positive and final results 2-3 days after the blood culture is positive. In this retrospective case-control study, cases were patients with polymicrobial BSIs, and controls were patients with monomicrobial BSIs. Each case was matched to three controls by age ±5 years (neonates ±5 days), admission date, and admitted wards.

Blood culture results of patients aged 0-86 years with polymicrobial and monomicrobial growth were first retrieved from the laboratory registry. Patients' information, including ages, sex, admission wards, date of admission, time of diagnosis of BSIs, date of discharge, comorbidity, and clinical outcomes (death or survival), were collected from clinical case notes. All cases and their matched controls with complete demographics and clinical information were included in the analysis. Only the first episode was included in cases where patients had two or more blood cultures positive with similar organisms. Patients with incomplete demographics and medical records were excluded from the analysis.

Microbiological methods for blood culture at MNH

Blood samples for culture were incubated in the BD BACTEC FX 200 at MNH, central pathology laboratory. Only one set of blood cultures was performed in adults and pediatrics. A set of BD BACTEC Plus Aerobic and Anaerobic/F Culture Vials (Becton Dickinson, BD) were processed for adults. For pediatrics, 1-3 mL of blood sample was collected in the BD BACTEC Peds PlusTM/F Culture Vials. Blood culture vials in BD BACTEC 200 were incubated for a maximum of five days before being regarded as a negative culture.

Positive blood cultures were Gram-stained and sub-cultured into sheep blood agar, chocolate agar, and MacConkey agar. Identification of bacteria was based on colonial morphology, Gram stain, and biochemical tests, including conventional API20E (BioMérieux, Marcy, I'Etoile, France) and Staphaurex (Remel Europe Ltd, Dartford, UK). Antimicrobial susceptibility testing was performed and interpreted according to clinical and laboratory standard institute (CLSI) guidelines [14].

Definition of terms

Polymicrobial BSI was defined as bacteremia with at least two different organisms from the same blood culture. The onset of BSI was defined as the day when the blood was drawn for culture that

subsequently produced positive pathogenic growth. Laboratory-confirmed BSIs was defined as blood culture with the growth of recognizable pathogens in patients with signs and symptoms of BSIs. Coagulase-negative staphylococci were considered a pathogen in immunosuppressed patients and neonates, in accordance with hospital blood culture standard operational procedure. *Micrococcus*, *Corynebacterium*, *Viridans* Group *streptococci*, and *Bacillus* species were considered as contaminants. The 30-day mortality was defined as death from any cause within 30 days of the onset of BSI. In-hospital mortality was defined as death from any cause during index hospitalization following diagnosis of BSI. The length of hospital stay was calculated from the date of diagnosis of BSIs. It excluded all patients with in-hospital mortality.

In addition, appropriate treatment was defined as empirical antimicrobial therapy with an antibiotic that has in vitro activity against the isolated pathogen based on susceptibility testing results.

Statistical analysis

Categorical variables were presented as frequency and percentages. A comparison of categorical variables between cases and controls was performed using Pearson chi-square. The median length of hospital stays among cases, and controls was compared using the Wilcoxon rank-sum test. Binary logistic regression compared outcomes of interest (30-day and in-hospital mortality) between cases and controls. All plausible biological variables (Admission ward, comorbidity, and appropriate treatment) with *p*-values < 0.20 on the bivariate analysis were included in the logistic regression modal for the multivariable analysis.

Multivariable logistic regression was performed to find an independent association of polymicrobial and monomicrobial BSIs to 30-day and in-hospital mortality. We adjusted for age, sex, admission ward, appropriate treatment, and comorbidity as potential confounders, defining confounders as factors that change the effect size by ≥10%.

We performed a sub-analysis for neonates only, where bivariate and multivariable logistic regression were performed. Independent associations between polymicrobial BSIs and outcomes of interest (30-day and in-hospital mortality) were adjusted for age, sex, admission ward, and comorbidity. All statistical analysis was performed using STATA version 16 (College Station, TX). A significance level of ≤0.05 was used, and all p-values refer to two-sided tests.

Ethics approval and consent to participate

Ethical approval was granted by the Muhimbili University of Health and Allied Sciences senate and publications committee Ref.No DA.25/111/01C/157. Permission to conduct the study was approved by Muhimbili National Hospital management. As the study was a retrospective study using identified patients, informed consent was waived by Muhimbili University of Health and Allied Sciences senate and publication committee. All methods were carried out under relevant guidelines and regulations in the declaration.

Results

Demographics and clinical characteristics

Between July 2021 and June 2022, 3398 blood cultures were processed at the microbiology laboratory of MNH. Overall, the proportion of BSI was 19% (642/3398). From 642 patients with BSI, we obtained 50 cases with polymicrobial BSI, matched to 150 controls with monomicrobial BSI in a ratio of 1:3. The age distributions among the cases and controls ranged from 1 day to 86 years, with majority of partipants being neonates. There were no significant differences in sex and comorbidities between polymicrobial BSI and monomicrobial BSI (Table 1).

Microbiology of bloodstream infections

Table 2 provides the microbiology for both polymicrobial and monomicrobial BSIs. The bacteria-causing polymicrobial and monomicrobial BSIs were similar, with the predominance of Gram-

negatives, although they varied in frequency. The most frequent bacteria in polymicrobial BSIs were *Klebsiella pneumoniae* 17% (17/100) and *Enterobacter* species 15% (15/100). Combinations of these two bacteria, as well as *S. aureus*/*Pseudomonas aeruginosa* and *Acinetobacter* species/CoNS, were also frequent, occurring in three instances each. In monomicrobial BSIs, *S. aureus* 17.33% (26/150), *Klebsiella pneumoniae* 16.67% (25/150), and *Acinetobacter* species 10% (15/150) were more prevalent (Table 2).

Antimicrobial susceptibility pattern

Overall, isolates were frequently resistant to multiple antibiotics tested, and 53% (130/243) were multidrug resistance. We observed that 77% (27/35) of *S. aureus* and 85% (25/29) of coagulase-negative Staphylococcus were resistant to cefoxitin, hence methicillin-resistant strains. All Enterobacterales were frequently resistant to all antibiotics tested except for meropenem and amikacin. Furthermore, *S. aureus* was less resistant to chloramphenicol (28%), clindamycin (38%), and trimethoprim-sulfamethoxazole (45%) (Table 3).

Outcomes in bloodstream infections

Overall, the 30-day mortality was 33.5% (67/200). The risk of mortality was higher in polymicrobial BSI compared to monomicrobial BSI, with an odd ratio (OR) of 1.83 (95% confidence interval (CI) 0.95-3.54), though the difference was not statistically significant (p = 0.07). However, after adjusting for possible confounders including sex, age, the admission ward, appropriate treatment, and comorbidity in a multivariable model, polymicrobial BSI independently predicted 30-day mortality, aOR 2.05, 95%CI 1.03-4.08), p = 0.04. Additionally, in-hospital mortality was 36% (72/200), with cases dying more frequently than controls (50% vs. 31.3%). We observed that polymicrobial BSI was a risk factor for in-hospital death based on univariable analysis crude odd ratio (cOR), 2.19, 95%CI 1.14-4.21, p = 0.02. We considered sex, age, the

admission ward, appropriate treatment, and comorbidity in the final multivariable analysis model as potential confounding variables. After adjusting for these variables, we found that polymicrobial BSIs adjusted odd ratio (aOR) 2.37, 95% CI 1.20-4.69, p = 0.01, had an independent association with in-hospital mortality (Table 4).

In sub-analyses involving only neonates, we found that polymicrobial BSI had an independent association with both 30-day mortality (aOR 4.02, 95% CI 1.32-12.32, p = 0.01) and in-hospital mortality (aOR 6.18, 95% CI 1.89-20.22, p = 0.003) after adjusting for, sex, appropriate treatment, and comorbidities. For all age groups and neonates, the median length of hospital stay post-BSIs was numerically longer in patients with polymicrobial than in those with monomicrobial BSIs, though the difference was not statistically significant (Table 5).

In a sub-analysis of non-neonates, we observed that polymicrobial BSI had no association with both in-hospital and 30-day mortality (Table 5).

Discussion

Our study demonstrates that polymicrobial BSIs are significantly associated with in-hospital mortality compared to monomicrobial BSIs in patients of all age groups. We observed significantly higher 30-day mortality in participants with polymicrobial BSI than those with monomicrobial BSI. We also found that patients with polymicrobial BSIs had a longer median length of hospital stay than patients with monomicrobial BSIs, but the differences were not statistically significant. A similar finding has been reported from previous studies, which found polymicrobial BSIs increase the risk of mortality [11, 15]. The difficulties in selecting appropriate empiric antimicrobials targeting multiple pathogens and delays in reporting blood culture results could impact our study's observed findings.

Most participants in this study received inappropriate treatment, which was more common in the polymicrobial BSI arm; however, we adjusted for inappropriate treatment in our analysis, and polymicrobial remained the main contributor to mortality. We could not account for the turn-around time of reporting polymicrobial BSI bacteriology results to clinicians as a possible contributor to the delay in initiation of treatment, possibly leading to increased mortality. Subsequently, ceftriaxone was the most commonly prescribed empiric antibiotic on review of clinical case notes, and most bacteria we isolated were resistant to this antibiotic. Our findings call for microbiologists and clinicians to be more vigilant when blood culture reveals polymicrobial BSI. We noted that the blood culture standard operating procedure does not detail how to communicate when polymicrobial BSIs are detected. On the other hand, the guidelines for treating sepsis consider only aerobic and anaerobic polymicrobial BSIs, and consideration is not given to aerobic-aerobic polymicrobial BSIs.

When we examined the neonates' risk factors for 30-day mortality, we found polymicrobial BSIs were an independent risk for mortality in neonates after adjusting for all possible confounders. The previous study in other settings has observed similar findings in neonates, where polymicrobial bloodstream infections were associated with 3 fold increased risk of mortality compared to monomicrobial bloodstream infection [10]. However, other studies in neonates have found comparable 30-day mortality between polymicrobial and monomicrobial BSIs [16, 17]. Immature immunity in neonates increases vulnerability to serious infections leading to an increased mortality risk. On the other hand, inappropriate antibiotic therapy compounded by overwhelming virulence from more than one pathogen would have led to the severity of illness in neonates, leading to the worse clinical outcomes observed.

Studies have reported that polymicrobial infections are associated with forming polymicrobial biofilms, which tend to increase pathogens' virulence, host immune evasion, and antibiotic resistance, hence poor treatment outcomes [18-20]. Polymicrobial BSI occurs more in neonates with severe underlying conditions and prolonged indwelling central venous catheters [16]. As central venous catheters were not commonly used at MNH during study time, the observed cases can be attributed to overwhelming illness.

We found that the median length of hospital stay was longer in patients with polymicrobial BSIs than in patients with monomicrobial BSIs. This could be due to the ineffectiveness of empiric antibiotic therapy since it was impossible for the prescribed empiric antibiotics to be effective in two different organisms. Therefore, the likelihood of receiving inappropriate treatment resulting in failure and prolonged hospital stays was obvious. Our findings are similar to those reported in the studies conducted in other settings [13, 21-23]. This finding calls for establishing a local cumulative antibiogram to guide empiric therapy in suspected bacteria infections, as it currently not existing in health facilities in the country.

The microbiology of polymicrobial BSIs was similar to that of monomicrobial infections in this study, with the predominant Gram-negative bacteria. This indicates that the sources for polymicrobial and monomicrobial BSIs are the same. Other studies examining the microbiological profiles of polymicrobial and monomicrobial BSIs have reported similar microbiology patterns in both cases [12, 17, 24]. However, depending on the variability of the population studied, the predominance of either Gram-positive or Gram-negative has been reported. On the other hand, we found that most bacteria causing BSIs in our study were MDR, which limits the available treatment options in our settings. The findings of this study emphasize the need for microbiology laboratories

to promptly perform and report antimicrobial susceptibility results to guide evidence-based antibiotics therapy.

One of the important caveats of our study was that it was a retrospective design dependent on recorded information from clinical case notes. Information like the severity of BSIs and the classification of BSIs (community onset versus hospital onset) was not documented in the clinical case notes, and this means missing data could introduce information bias. However, missing end-point outcomes would be non-differentially distributed evenly across polymicrobial and monomicrobial BSIs. Furthermore, we performed multivariable analysis to control for possible confounding variables associated with mortality. Another important limitation of our study is that we could not assess the risk factors for polymicrobial BSIs because of the missing information. Understanding risk factors for polymicrobial BSIs would be critical to controlling and preventing infections before they even occur. However, our findings call for microbiologists and clinicians to manage polymicrobial BSIs because of the associated risk aggressively.

Conclusion

In neonates, polymicrobial BSI is an independent risk for 30-day and in-hospital mortality and . Overall, the median length of hospital stay was longer in patients with polymicrobial BSI than those with monomicrobial BSI. These findings highlight the need for clinicians to be more aggressive when polymicrobial BSI is detected. The laboratory should also prompt notify clinician when polymicrobial growth is detected in blood culture.

Acknowledgements

The authors acknowledge the members of the bacteriology section at the central pathology laboratory, MNH.

Authors' contributions

JM conceived the study, performed analysis, and drafted the manuscript. AM and MM collected data. AJ, FM, MK, HN, and MVM revised the manuscript. All authors read and approved the manuscript.

References

1. Prasad N, Murdoch DR, Reyburn H, Crump JA. Etiology of Severe Febrile Illness in Low- and Middle-Income Countries: A Systematic Review. PLoS One. 2015;10(6):e0127962. Epub 20150630. doi: 10.1371/journal.pone.0127962. PubMed PMID: 26126200; PubMed Central PMCID: PMCPMC4488327.
2. Marchello CS, Dale AP, Pisharody S, Rubach MP, Crump JA. A Systematic Review and Meta-analysis of the Prevalence of Community-Onset Bloodstream Infections among Hospitalized Patients in Africa and Asia. Antimicrob Agents Chemother. 2019;64(1). Epub 20191220. doi: 10.1128/aac.01974-19. PubMed PMID: 31636071; PubMed Central PMCID: PMCPMC7187598.
3. Moyo SJ, Manyahi J, Blomberg B, Tellevik MG, Masoud NS, Aboud S, et al. Bacteraemia, Malaria, and Case Fatality Among Children Hospitalized With Fever in Dar es Salaam, Tanzania. Front Microbiol. 2020;11:2118. Epub 20200910. doi: 10.3389/fmicb.2020.02118. PubMed PMID: 33013772; PubMed Central PMCID: PMCPMC7511546.
4. Seni J, Mwakyoma AA, Mashuda F, Marando R, Ahmed M, DeVinney R, et al. Deciphering risk factors for blood stream infections, bacteria species and antimicrobial resistance profiles among children under five years of age in North-Western Tanzania: a multicentre study in a cascade of referral health care system. BMC Pediatr. 2019;19(1):32. Epub 20190126. doi: 10.1186/s12887-019-1411-0. PubMed PMID: 30684964; PubMed Central PMCID: PMCPMC6347777.

5. Manyahi J, Kibwana U, Mgimba E, Majigo M. Multi-drug resistant bacteria predict mortality in bloodstream infection in a tertiary setting in Tanzania. PLoS One. 2020;15(3):e0220424. Epub 20200304. doi: 10.1371/journal.pone.0220424. PubMed PMID: 32130227; PubMed Central PMCID: PMCPMC7055912.
6. Godfrey E, Majaliwa E, Assenga EN. Aetiology, antimicrobial susceptibility and outcome of children with sepsis, admitted at Muhimbili National Hospital, Dar es Salaam. Pan Afr Med J. 2022;42:167. Epub 20220701. doi: 10.11604/pamj.2022.42.167.29969. PubMed PMID: 36187027; PubMed Central PMCID: PMCPMC9482214.
7. Kortz TB, Sawe HR, Murray B, Enanoria W, Matthay MA, Reynolds T. Clinical Presentation and Outcomes among Children with Sepsis Presenting to a Public Tertiary Hospital in Tanzania. Front Pediatr. 2017;5:278. Epub 20171222. doi: 10.3389/fped.2017.00278. PubMed PMID: 29312910; PubMed Central PMCID: PMCPMC5743673.
8. Blomberg B, Manji KP, Urassa WK, Tamim BS, Mwakagile DS, Jureen R, et al. Antimicrobial resistance predicts death in Tanzanian children with bloodstream infections: a prospective cohort study. BMC Infect Dis. 2007;7:43. Epub 20070522. doi: 10.1186/1471-2334-7-43. PubMed PMID: 17519011; PubMed Central PMCID: PMCPMC1891109.
9. Blomberg B, Jureen R, Manji KP, Tamim BS, Mwakagile DS, Urassa WK, et al. High rate of fatal cases of pediatric septicemia caused by gram-negative bacteria with extended-spectrum beta-lactamases in Dar es Salaam, Tanzania. J Clin Microbiol. 2005;43(2):745-9. doi: 10.1128/jcm.43.2.745-749.2005. PubMed PMID: 15695674; PubMed Central PMCID: PMCPMC548071.

10. Pammi M, Zhong D, Johnson Y, Revell P, Versalovic J. Polymicrobial bloodstream infections in the neonatal intensive care unit are associated with increased mortality: a case-control study. BMC Infect Dis. 2014;14:390. Epub 20140714. doi: 10.1186/1471-2334-14-390. PubMed PMID: 25022748; PubMed Central PMCID: PMCPMC4226990.

11. Zhang L, Liu ZY, Xu YC, Li TS, Yang QW, Wang AX. [The microbiological and clinical analysis of bloodstream infections with identifiable sources]. Zhonghua Nei Ke Za Zhi. 2012;51(5):366-70. PubMed PMID: 22883335.

12. Lin JN, Lai CH, Chen YH, Chang LL, Lu PL, Tsai SS, et al. Characteristics and outcomes of polymicrobial bloodstream infections in the emergency department: A matched case-control study. Acad Emerg Med. 2010;17(10):1072-9. doi: 10.1111/j.1553-2712.2010.00871.x. PubMed PMID: 21040108.

13. Zheng C, Zhang S, Chen Q, Zhong L, Huang T, Zhang X, et al. Clinical characteristics and risk factors of polymicrobial *Staphylococcus aureus* bloodstream infections. Antimicrob Resist Infect Control. 2020;9(1):76. Epub 20200527. doi: 10.1186/s13756-020-00741-6. PubMed PMID: 32460851; PubMed Central PMCID: PMCPMC7254655.

14. Clinical and Laboratory Standards Institute (2018) Performance standards for antimicrobial susceptibility testing; twenty-eighth informational supplement CLSI document M100–S20. Clinical and Laboratory Standards Institute W, PA.

15. Xu J, Yuan Y, Wang B, Zhang Q, Wang J, Wang S, et al. Microbiological Analysis and Mortality Risk Factors in Patients with Polymicrobial Bloodstream Infections. Infect Drug Resist. 2023;16:3917-27. Epub 20230620. doi: 10.2147/idr.S412669. PubMed PMID: 37361937; PubMed Central PMCID: PMCPMC10290474.

16. Bizzarro MJ, Dembry LM, Baltimore RS, Gallagher PG. Matched case-control analysis of polymicrobial bloodstream infection in a neonatal intensive care unit. Infect Control Hosp Epidemiol. 2008;29(10):914-20. doi: 10.1086/591323. PubMed PMID: 18808341.
17. Tsai MH, Chu SM, Hsu JF, Lien R, Huang HR, Chiang MC, et al. Polymicrobial bloodstream infection in neonates: microbiology, clinical characteristics, and risk factors. PLoS One. 2014;9(1):e83082. Epub 20140114. doi: 10.1371/journal.pone.0083082. PubMed PMID: 24454692; PubMed Central PMCID: PMCPMC3891628.
18. Nabb DL, Song S, Kluthe KE, Daubert TA, Luedtke BE, Nuxoll AS. Polymicrobial Interactions Induce Multidrug Tolerance in *Staphylococcus aureus* Through Energy Depletion. Front Microbiol. 2019;10:2803. Epub 20191205. doi: 10.3389/fmicb.2019.02803. PubMed PMID: 31866973; PubMed Central PMCID: PMCPMC6906149.
19. Little W, Black C, Smith AC. Clinical Implications of Polymicrobial Synergism Effects on Antimicrobial Susceptibility. Pathogens. 2021;10(2). Epub 20210201. doi: 10.3390/pathogens10020144. PubMed PMID: 33535562; PubMed Central PMCID: PMCPMC7912749.
20. Tay WH, Chong KK, Kline KA. Polymicrobial-Host Interactions during Infection. J Mol Biol. 2016;428(17):3355-71. Epub 20160508. doi: 10.1016/j.jmb.2016.05.006. PubMed PMID: 27170548.
21. Sutter D, Stagliano D, Braun L, Williams F, Arnold J, Ottolini M, et al. Polymicrobial bloodstream infection in pediatric patients: risk factors, microbiology, and antimicrobial management. Pediatr Infect Dis J. 2008;27(5):400-5. doi: 10.1097/INF.0b013e31816591be. PubMed PMID: 18398386.

22. Song F, Zhang K, Huang J, Qian Z, Zhou H, Cai J, et al. Clinical Characteristics, Risk Factors, and Outcomes of Patients with Polymicrobial *Klebsiella pneumoniae* Bloodstream Infections. Biomed Res Int. 2021;2021:6619911. Epub 20210619. doi: 10.1155/2021/6619911. PubMed PMID: 34239928; PubMed Central PMCID: PMCPMC8235985.

23. Sancho S, Artero A, Zaragoza R, Camarena JJ, González R, Nogueira JM. Impact of nosocomial polymicrobial bloodstream infections on the outcome in critically ill patients. Eur J Clin Microbiol Infect Dis. 2012;31(8):1791-6. Epub 20111214. doi: 10.1007/s10096-011-1503-8. PubMed PMID: 22167257.

24. Goldman S, Itshaki O, Shochat T, Gafter-Gvili A, Yahav D, Rubinovitch B, et al. Risk Factors and Outcome of Polymicrobial Bacteremia: A Retrospective Cohort Study. Isr Med Assoc J. 2020;22(5):279-84. PubMed PMID: 32378818.

Table 1: Demographic-clinical characteristics of the participants

Variable	Monomicrobial BSIs(N=150)	Polymicrobial BSIs (N=50)	*p*-value
Age (median, IQR)	1 (1-40)	1 (1-40)	
<1month	69	23	
<1 year	18	6	
2–16 year	9	3	
>17 years	54	18	
Sex, Male	75 (50%)	26 (52%)	0.8
Ward, ICU	90 (60%)	31 (62%)	0.8
Comorbidity, Yes	68 (45.3%)	22 (44%)	0.87
Appropriate treatment, Yes	72 (48%)	18 (36%)	0.14

IQR- Interquartile ratio; ICU-intensive care unit

Table 2: Bacterial isolates from monomicrobial and polymicrobial bloodstream infections

Bacteria	Monomicrobial BSI N(%)	Polymicrobial BSI N(%)
K. pneumoniae	25 (16.67)	17 (17.00)
E. coli	17(11.33)	7 (7.00)
Enterobacter spp	13(8.67)	15(15.00)
*Other Enterobacterales	13 (8.67)	16 (16.00)
Acinetobacter spp	15 (15)	9 (9.00)
P. aeruginosa	14 (9.33)	8 (8.00)
[#]Non-Enterobacterales	4 (2.67)	6(6.00)
S. aureus	26 (17.33)	9(9.00)
CoNS	20 (13.33)	9(9.00)
Streptococcus spp	2(1.33)	3(3.00)
Candida spp	1 (0.67)	1 (1.00)
**Total**	**150**	**100**

*Other Enterobacterales: *Morganella morganii*, *Serratia mercescens*, *Providencia* spp, *Citrobacter* species [#]Non-Enterobacterales: *Aeromonas* spp, *Chromobacteria violaceum*; CoNS:Coagulase negative Staphylococcus

Table 3: Antimicrobial susceptibility patterns of bacteria from bloodstream infections

	Percent Resistance															
**Bacteria (n)**	**AMP**	**AMC**	**TZP**	**CRO**	**CAZ**	**FEP**	**MEM**	**AMK**	**CN**	**CIP**	**CHL**	**SXT**	**P**	**FOX**	**ERY**	**CLI**
K. pneumoniae (42)	100	80	75	72	71	76	44	39	61	69	68	59				
E. coli (24)	92	81	75	80	74	90	26	44	53	76	36	100				
Enterobacter spp (28)	100	89	60	76	81	74	79	36	70	79	69	70				
*Other Enterobacterales (29)	84	75	38	84	52	87	45	38	60	58	53	69				
Acinetobacter spp (24)			70	89	59	68	57	37	65	50	46	55				
P. aeruginosa (22)			58		39	31	24	32	58	47						
#Non-Enterobacterales (10)			63		33	44	0	22	56	22	14	67				
S. aureus (35)									60	58	28	45	100	77	80	38
CoNS (29)									62	94	16	90	100	85	89	90
**Overall**	**91**	**79**	**64**	**80**	**64**	**72**	**43**	**36**	**62**	**64**	**48**	**67**	**100**	**81**	**84**	**64**

AMK-Amikacin, AMC-Amoxycillin-clavulanic acid, AMP-Ampicillin, FOX-Cefoxitin, CAZ-Ceftazidime, CRO-Ceftriaxone, CHL-Chloramphenicol, CIP-Ciprofloxacin, CLI-Clindamycin, ERY-Erythromycin, GEN-Gentamicin, MEM-Meropenem, TZP-Tazobactam-piperacillin, P-Penicillin, SXTTrimethoprim-sulfamethoxazole. Other Enterobacterales: *Morganella morganii*, *Serratia mercescens*, *Providencia* spp, *Citrobacter* species #Non-Enterobacterales: *Aeromonas* spp, *Chromobacteria violaceum*.
CoNS:Coagulase negative Staphylococcus

Table 4. Multivariable analysis assessing the association between BSIs and mortality

Outcome	Monomicrobial BSIs	Polymicrobial BSIs	cOR, 95%CI, *p*-value	aOR, 95%CI, *p*-value
**All patients**	**N=150**	**N=50**		
30-days mortality (%)	30	44	1.83 (0.95-3.54), 0.07	2.05, (1.03-4.08), 0.04
In-hospital mortality (%)	31.3	50	2.19 (1.14-4.21), 0.02	2.37, (1.20-4.69), 0.01
**Neonates only**	**N=69**	**N=23**		
30-days mortality (%)	42.03	65.22	2.78, 1.04-7.39, 0.04	4.02, 1.32-12.23, 0.01
In-hospital mortality (%)	42.03	73.91	4.20, 1.48-11.92, 0.007	6.18, 1.89-20.22, 0.003
**Non-neonates**	**N=81**	**N=27**		
30-days mortality (%)	19.75	25.93	1.42, 0.51-3.94, 0.50	1.55, 0.55- 4.41, 0.59
In-hospital mortality (%)	22.22	29.63	1.47, 0.55-3.91, 0.45	1.59, 0.58 - 4.36, 0.36

BSI: Bloodstream infections, cOR: crude odd ratio, aOR: adjustable odd ratio, CI: Confidence interval

Table 5: Length of hospital stay post-bloodstream infections

Outcome	Monomicrobial BSIs	Polymicrobial BSIs	*p*-value
**All patients**	**N=105**	**N=28**	
LOS (median, IQR)	13 (5-24)	26 (6-37)	0.2
**Neonates only**	**N=43**	**N=8**	
LOS (median, IQR)	13 (6-32)	32 (21.5-62)	0.07

LOS: Length of hospital stay, IQR: Interquartile range, BSI: Bloodstream infections

Polymicrobial bloodstream infections a risk factor for mortality in neonates at the national hospital, Tanzania: A case-control study

Authors: Joel Manyahi[1*], Agricola Joachim[1], Frank Msafiri[1], Mary Migiro[1], Anthon Mwingwa[1], Mabula Kasubi[2], Helga Naburi[3], Mtebe Venance Majigo[1]

Affiliations:

[1]Department of Microbiology and Immunology, Muhimbili University of Health and Allied Sciences, Dar es Salaam, Tanzania.

[2]Muhimbili National Hospital, Dar es Salaam, Tanzania.

[3]Department of Pediatrics and Child Health, Muhimbili University of Health and Allied Sciences, Dar es Salaam, Tanzania.

Email:

Joel Manyahi (manyahijoel@yahoo.com)

Agricola Joachim (agricolaj@yahoo.com)

Frank Msafiri (frankmbulinyingi@yahoo.com)

Field Code Changed

Mary Migiro (marymigiro03@gmail.com)

Anthon Mwingwa (anthonymwingwa@gmail.com)

Mabula Kasubi (Mkasubi68@gmail.com)

Field Code Changed

Helga Naburi (hnaburi2000@yahoo.com

Mtebe **Venance** Majigo (mmajigo@gmail.com)

Corresponding Author*

Joel Manyahi, Muhimbili University of Health and Allied Sciences, P.O. Box 65001, Dar es Salaam, Tanzania

Mobile Number +255712251709

Keywords: Polymicrobial, bloodstream infections, mortality, Tanzania

Abstract

Background: Polymicrobial bloodstream infections (BSI) are difficult to treat since empiric antibiotics are frequently less effective against multiple pathogens. The study aimed to compare outcomes in patients with polymicrobial and monomicrobial BSIs.

Methods: The study was a retrospective case-control design conducted at Muhimbili National Hospital for data processed between July 2021 and June 2022. Cases were patients with polymicrobial BSI, and controls had monomicrobial BSI. Each case was matched to three controls by age, admitting ward, and duration of admission. Logistic regression was performed to determine independent risk factors for in-hospital and 30-day mortality.

Results: Fifty patients with polymicrobial BSI and 150 with monomicrobial BSI were compared: the two had no significant differences in sex and comorbidities. The most frequent bacteria in polymicrobial BSI were *Klebsiella pneumoniae* 17% (17/100) and *Enterobacter* species 15% (15/100). In monomicrobial BSI, *S. aureus* 17.33% (26/150), *Klebsiella pneumoniae* 16.67% (25/150), and *Acinetobacter* species 15% (15/150) were more prevalent. Overall, isolates were frequently resistant to multiple antibiotics tested, and 52% (130/250) were multidrug resistance. The 30-day and in-hospital mortality were 33.5% (67/200) and 36% (72/200), respectively. On multivariable analysis, polymicrobial BSIs were independent risk factors for both in-hospital mortality (aOR 2.37, 95%CI 1.20-4.69, p = 0.01) and 30-day mortality (aOR 2.05, 95%CI 1.03-4.08), p = 0.04). In sub-analyses involving only neonates, polymicrobial BSI was an independent risk factor for both 30-day mortality (aOR 3.13, 95%CI 1.07-9.10, p = 0.04) and in-hospital mortality (aOR 5.08, 95%CI 1.60-16.14, p = 0.006). Overall, the median length of hospital stay post-BSIs was numerically longer in patients with polymicrobial BSIs.

Conclusion: Overall, mortality was significantly frequent in patients with polymicrobial BSI. Patients with polymicrobial BSI stay long at the hospital than monomicrobial BSI. These findings call for clinicians to be more aggressive in managing polymicrobial BSI.

Background

Bloodstream infections (BSI) in low-middle-income countries are steadily associated with poor treatment outcomes, with the rising concern associated with antibiotic-resistant bacteria. Bacteremia has been reported in 10–13% of inpatients with severe febrile illness in East and West Africa, with an estimated case fatality rate of 12% [1, 2].

Following the reduction of malaria, a frequent cause of febrile illness in Tanzania, BSIs remain a common cause of hospitalization in children with febrile illness. In Tanzania, BSIs is detected in 11–14% of children with severe febrile illness [3, 4]. Studies have shown that BSIs in adults and children result in worse outcomes, such as high mortality and length of hospital stay [5-8]. In addition, increasing evidence of antibiotic-resistant bacteria, including methicillin-resistant *Staphylococcus aureus* (MRSA), extended-spectrum beta-lactamases (ESBL), and multidrug-resistant (MDR) bacteria in BSIs, has been reported in Tanzania [3-5, 9].

Studies in Tanzania have reported that BSIs due to antibiotic-resistant bacteria had a significantly higher risk of death than those with antibiotic susceptible bacteria [5, 8, 9]. Mortality in children with ESBL septicemia was approximately 2-fold higher than in non-ESBL septicemia [9]. Our recent study found that patients with MDR BSIs (aOR = 15) had a higher risk of dying than those with non-MDR BSIs [5]. However, studies still need to evaluate the outcomes of polymicrobial BSIs in Tanzania thoroughly.

Polymicrobial BSIs are frequently difficult to treat, and choosing the appropriate empiric antibiotics effective for more than one pathogen is more challenging. It is, therefore, vital to

promptly identify the pathogens causing polymicrobial BSIs and administer the proper empiric antibiotics/drugs. In addition, studies on adults and children from other settings have demonstrated that mortality attributable to polymicrobial BSIs is higher than in monomicrobial BSIs [10-13]. Therefore, we carried out this retrospective case-control study at Muhimbili National Hospital (MNH) to ascertain the impact of polymicrobial BSIs on patient outcomes. A better understanding of the risk factors for worse outcomes in patients with BSIs would improve clinical outcomes for patients.

Materials and methods

Study setting and design

This was a retrospective case-control study for the data processed between July 2021 and June 2022 at MNH, Dar es Salaam, Tanzania. The data for this study were accessed and collected between July 1, 2022, and September 30, 2022. MNH is a tertiary-level hospital in Tanzania and serves as a teaching hospital for Muhimbili University of Health and Allied Sciences (MUHAS). It has a 1,500-bed capacity and attends up to 2,000 outpatients daily. The hospital has an infection control policy and an infection prevention and control (IPC) unit. Furthermore, blood culture sample in all wards is collected under aseptic condition and transferred to the laboratory within one hour of collection. Under ideal situations, the laboratory reporting system allows to flag clinicians with preliminary results immediately when the blood culture flag-positive and final results 2-3 days after the blood culture is positive. In this retrospective case-control study, cases were patients with polymicrobial BSIs, and controls were patients with monomicrobial BSIs. Each case was matched to three controls by age ±5 years (neonates ±5 days), admission date, and admitted wards.

Blood culture results of patients aged 0-86 years with polymicrobial and monomicrobial growth were first retrieved from the laboratory registry. Patients' information, including ages, sex, admission wards, date of admission, time of diagnosis of BSIs, date of discharge, comorbidity, and clinical outcomes (death or survival), were collected from clinical case notes. All cases and their matched controls with complete demographics and clinical information were included in the analysis. Only the first episode was included in cases where patients had two or more blood cultures positive with similar organisms. Patients with incomplete demographics and medical records were excluded from the analysis.

Microbiological methods for blood culture at MNH

Blood samples for culture were incubated in the BD BACTEC FX 200 at MNH, central pathology laboratory. Only one set of blood cultures was performed in adults and pediatrics. A set of BD BACTEC Plus Aerobic and Anaerobic/F Culture Vials (Becton Dickinson, BD) were processed for adults. For pediatrics, 1-3 mL of blood sample was collected in the BD BACTEC Peds PlusTM/F Culture Vials. Blood culture vials in BD BACTEC 200 were incubated for a maximum of five days before being regarded as a negative culture.

Positive blood cultures were Gram-stained and sub-cultured into sheep blood agar, chocolate agar, and MacConkey agar. Identification of bacteria was based on colonial morphology, Gram stain, and biochemical tests, including conventional API20E (BioMérieux, Marcy, I'Etoile, France) and Staphaurex (Remel Europe Ltd, Dartford, UK). Antimicrobial susceptibility testing was performed and interpreted according to clinical and laboratory standard institute (CLSI) guidelines [14].

Definition of terms

Polymicrobial BSI was defined as bacteremia with at least two different organisms from the same blood culture. The onset of BSI was defined as the day when the blood was drawn for culture that

subsequently produced positive pathogenic growth. Laboratory-confirmed BSIs was defined as blood culture with the growth of recognizable pathogens in patients with signs and symptoms of BSIs. Coagulase-negative staphylococci were considered a pathogen in immunosuppressed patients and neonates, in accordance with hospital blood culture standard operational procedure. *Micrococcus*, *Corynebacterium*, *Viridans* Group *streptococci*, and *Bacillus* species were considered as contaminants. The 30-day mortality was defined as death from any cause within 30 days of the onset of BSI. In-hospital mortality was defined as death from any cause during index hospitalization following diagnosis of BSI. The length of hospital stay was calculated from the date of diagnosis of BSIs. It excluded all patients with in-hospital mortality.

In addition, appropriate treatment was defined as empirical antimicrobial therapy with an antibiotic that has in vitro activity against the isolated pathogen based on susceptibility testing results.

Statistical analysis

Categorical variables were presented as frequency and percentages. A comparison of categorical variables between cases and controls was performed using Pearson chi-square. The median length of hospital stays among cases, and controls was compared using the Wilcoxon rank-sum test. Binary logistic regression compared outcomes of interest (30-day and in-hospital mortality) between cases and controls. All plausible biological variables (Admission ward, comorbidity, and appropriate treatment) with *p*-values < 0.20 on the bivariate analysis were included in the logistic regression modal for the multivariable analysis.

Multivariable logistic regression was performed to find an independent association of polymicrobial and monomicrobial BSIs to 30-day and in-hospital mortality. We adjusted for age, sex, admission ward, appropriate treatment, and comorbidity as potential confounders, defining confounders as factors that change the effect size by $\geq$10%.

We performed a sub-analysis for neonates only, where bivariate and multivariable logistic regression were performed. Independent associations between polymicrobial BSIs and outcomes of interest (30-day and in-hospital mortality) were adjusted for age, sex, admission ward, and comorbidity. All statistical analysis was performed using STATA version 16 (College Station, TX). A significance level of ≤0.05 was used, and all p-values refer to two-sided tests.

Ethics approval and consent to participate

Ethical approval was granted by the Muhimbili University of Health and Allied Sciences senate and publications committee Ref.No DA.25/111/01C/157. Permission to conduct the study was approved by Muhimbili National Hospital management. As the study was a retrospective study using identified patients, informed consent was waived by Muhimbili University of Health and Allied Sciences senate and publication committee. All methods were carried out under relevant guidelines and regulations in the declaration.

Results

Demographics and clinical characteristics

Between July 2021 and June 2022, 3398 blood cultures were processed at the microbiology laboratory of MNH. Overall, the proportion of BSI was 19% (642/3398). From 642 patients with BSI, we obtained 50 cases with polymicrobial BSI, matched to 150 controls with monomicrobial BSI in a ratio of 1:3. The age distributions among the cases and controls ranged from 1 day to 86 years, with majority of partipants being neonates. There were no significant differences in sex and comorbidities between polymicrobial BSI and monomicrobial BSI (Table 1).

Microbiology of bloodstream infections

Table 2 provides the microbiology for both polymicrobial and monomicrobial BSIs. The bacteria-causing polymicrobial and monomicrobial BSIs were similar, with the predominance of Gram-

negatives, although they varied in frequency. The most frequent bacteria in polymicrobial BSIs were *Klebsiella pneumoniae* 17% (17/100) and *Enterobacter* species 15% (15/100). Combinations of these two bacteria, as well as *S. aureus*/*Pseudomonas aeruginosa* and *Acinetobacter* species/CoNS, were also frequent, occurring in three instances each. In monomicrobial BSIs, *S. aureus* 17.33% (26/150), *Klebsiella pneumoniae* 16.67% (25/150), and *Acinetobacter* species 10~~5~~% (15/150) were more prevalent (Table 2).

Antimicrobial susceptibility pattern

Overall, isolates were frequently resistant to multiple antibiotics tested, and 53~~2~~% (130/243~~50~~) were multidrug resistance. We observed that 77% (27/35) of *S. aureus* and 85% (25/29) of coagulase-negative Staphylococcus were resistant to cefoxitin, hence methicillin-resistant strains. All Enterobacterales were frequently resistant to all antibiotics tested except for meropenem and amikacin. Furthermore, *S. aureus* was less resistant to chloramphenicol (28%), clindamycin (38%), and trimethoprim-sulfamethoxazole (45%) (Table 3).

Outcomes in bloodstream infections

Overall, the 30-day mortality was 33.5% (67/200). The risk of mortality was higher in polymicrobial BSI compared to monomicrobial BSI, with an odd ratio (OR) of 1.83 (95% confidence interval (CI) 0.95-3.54), though the difference was not statistically significant (p = 0.07). However, after adjusting for possible confounders including sex, age, the admission ward, appropriate treatment, and comorbidity in a multivariable model, polymicrobial BSI independently predicted 30-day mortality, aOR 2.05, 95%CI 1.03-4.08), p = 0.04. Additionally, in-hospital mortality was 36% (72/200), with cases dying more frequently than controls (50% vs. 31.3%). We observed that polymicrobial BSI was a risk factor for in-hospital death based on univariable analysis crude odd ratio (cOR), 2.19, 95%CI 1.14-4.21, p = 0.02. We considered sex, age, the

admission ward, appropriate treatment, and comorbidity in the final multivariable analysis model as potential confounding variables. After adjusting for these variables, we found that polymicrobial BSIs adjusted odd ratio (aOR) 2.37, 95% CI 1.20-4.69, p = 0.01, had an independent association with in-hospital mortality (Table 4).

In sub-analyses involving only neonates, we found that polymicrobial BSI had an independent association with both 30-day mortality (aOR 4.02, 95% CI 1.32-12.32, p = 0.01) and in-hospital mortality (aOR 6.18, 95% CI 1.89-20.22, p = 0.003) after adjusting for, sex, appropriate treatment, and comorbidities. For all age groups and neonates, the median length of hospital stay post-BSIs was numerically longer in patients with polymicrobial than in those with monomicrobial BSIs, though the difference was not statistically significant (Table 5).

In a sub-analysis of non-neonates, we observed that polymicrobial BSI had no association with both in-hospital and 30-day mortality (Table 5).

Formatted: Font: Times New Roman, 12 pt

Formatted: Left

Discussion

Our study demonstrates that polymicrobial BSIs are significantly associated with in-hospital mortality compared to monomicrobial BSIs in patients of all age groups. We observed significantly higher 30-day mortality in participants with polymicrobial BSI than those with monomicrobial BSI. We also found that patients with polymicrobial BSIs had a longer median length of hospital stay than patients with monomicrobial BSIs, but the differences were not statistically significant. A similar finding has been reported from previous studies, which found polymicrobial BSIs increase the risk of mortality [11, 15] [11-13, 16-19]. The difficulties in selecting appropriate

empiric antimicrobials targeting multiple pathogens and delays in reporting blood culture results could impact our study's observed findings.

Most participants in this study received inappropriate treatment, which was more common in the polymicrobial BSI arm; however, we adjusted for inappropriate treatment in our analysis, and polymicrobial remained the main contributor to mortality. We could not account for the turn-around time of reporting polymicrobial BSI bacteriology results to clinicians as a possible contributor to the delay in initiation of treatment, possibly leading to increased mortality. Subsequently, ceftriaxone was the most commonly prescribed empiric antibiotic on review of clinical case notes, and most bacteria we isolated were resistant to this antibiotic.

Our findings call for microbiologists and clinicians to be more vigilant when blood culture reveals polymicrobial BSI. We noted that the blood culture standard operating procedure does not detail how to communicate when polymicrobial BSIs are detected. On the other hand, the guidelines for treating sepsis consider only aerobic and anaerobic polymicrobial BSIs, and consideration is not given to aerobic-aerobic polymicrobial BSIs.

Formatted: Space After: 12 pt

Formatted: Justified, Space After: 12 pt

When we examined the ~~participants~~neonates’~~'~~ risk factors for 30-day mortality, we found polymicrobial BSIs were an independent risk for mortality in neonates after adjusting for all possible confounders. The previous study in other settings has observed similar findings in neonates, where polymicrobial bloodstream infections were associated with 3 fold increased risk of mortality compared to monomicrobial bloodstream infection [10]. However, other studies in neonates have found comparable 30-day mortality between polymicrobial and monomicrobial BSIs [16, 17]. Immature immunity in neonates increases vulnerability to serious infections leading

to an increased mortality risk. On the other hand, inappropriate antibiotic therapy compounded by overwhelming virulence from more than one pathogen would have led to the severity of illness in neonates, leading to the worse clinical outcomes observed.

Studies have reported that polymicrobial infections are associated with forming polymicrobial biofilms, which tend to increase pathogens' virulence, host immune evasion, and antibiotic resistance, hence poor treatment outcomes [18-20]. Polymicrobial BSI occurs more in neonates with severe underlying conditions and prolonged indwelling central venous catheters [16]. As central venous catheters were not commonly used at MNH during study time, the observed cases can be attributed to overwhelming illness.

We found that the median length of hospital stay was longer in patients with polymicrobial BSIs than in patients with monomicrobial BSIs. This could be due to the ineffectiveness of empiric antibiotic therapy since it was impossible for the prescribed empiric antibiotics to be effective in two different organisms. Therefore, the likelihood of receiving inappropriate treatment resulting in failure and prolonged hospital stays was obvious. Our findings are similar to those reported in the studies conducted in other settings [13, 21-23]. This finding calls for establishing a local cumulative antibiogram to guide empiric therapy in suspected bacteria infections, as it currently not existing in health facilities in the country.

The microbiology of polymicrobial BSIs was similar to that of monomicrobial infections in this study, with the predominant Gram-negative bacteria. This indicates that the sources for polymicrobial and monomicrobial BSIs are the same. Other studies examining the microbiological profiles of polymicrobial and monomicrobial BSIs have reported similar microbiology patterns in both cases [12, 17, 24]. However, depending on the variability of the population studied, the predominance of either Gram-positive or Gram-negative has been reported. On the other hand, we

found that most bacteria causing BSIs in our study were MDR, which limits the available treatment options in our settings. The findings of this study emphasize the need for microbiology laboratories to promptly perform and report antimicrobial susceptibility results to guide evidence-based antibiotics therapy.

One of the important caveats of our study was that it was a retrospective design dependent on recorded information from clinical case notes. Information like the severity of BSIs and the classification of BSIs (community onset versus hospital onset) was not documented in the clinical case notes, and this means missing data could introduce information bias. However, missing end-point outcomes would be non-differentially distributed evenly across polymicrobial and monomicrobial BSIs. Furthermore, we performed multivariable analysis to control for possible confounding variables associated with mortality. Another important limitation of our study is that we could not assess the risk factors for polymicrobial BSIs because of the missing information. Understanding risk factors for polymicrobial BSIs would be critical to controlling and preventing infections before they even occur. However, our findings call for microbiologists and clinicians to manage polymicrobial BSIs because of the associated risk aggressively.

Conclusion

~~In hospital mortality was significantly more frequent in patients with polymicrobial BSI than monomicrobial BSI.~~ In neonates, polymicrobial BSI is~~was~~ an independent risk for 30-day and in-hospital mortality and . Overall, the median length of hospital stay was longer in patients with polymicrobial BSI than those with monomicrobial BSI. These findings highlight the need for clinicians to be more aggressive when polymicrobial BSI is detected. The laboratory should also prompt notify clinician when polymicrobial growth is detected in blood culture.

Acknowledgements

The authors acknowledge the members of the bacteriology section at the central pathology laboratory, MNH.

Authors' contributions

JM conceived the study, performed analysis, and drafted the manuscript. AM and MM collected data. AJ, FM, MK, HN, and MVM revised the manuscript. All authors read and approved the manuscript.

References

1. Prasad N, Murdoch DR, Reyburn H, Crump JA. Etiology of Severe Febrile Illness in Low- and Middle-Income Countries: A Systematic Review. PLoS One. 2015;10(6):e0127962. Epub 20150630. doi: 10.1371/journal.pone.0127962. PubMed PMID: 26126200; PubMed Central PMCID: PMCPMC4488327.
2. Marchello CS, Dale AP, Pisharody S, Rubach MP, Crump JA. A Systematic Review and Meta-analysis of the Prevalence of Community-Onset Bloodstream Infections among Hospitalized Patients in Africa and Asia. Antimicrob Agents Chemother. 2019;64(1). Epub 20191220. doi: 10.1128/aac.01974-19. PubMed PMID: 31636071; PubMed Central PMCID: PMCPMC7187598.
3. Moyo SJ, Manyahi J, Blomberg B, Tellevik MG, Masoud NS, Aboud S, et al. Bacteraemia, Malaria, and Case Fatality Among Children Hospitalized With Fever in Dar es Salaam, Tanzania. Front Microbiol. 2020;11:2118. Epub 20200910. doi: 10.3389/fmicb.2020.02118. PubMed PMID: 33013772; PubMed Central PMCID: PMCPMC7511546.
4. Seni J, Mwakyoma AA, Mashuda F, Marando R, Ahmed M, DeVinney R, et al. Deciphering risk factors for blood stream infections, bacteria species and antimicrobial resistance profiles among children under five years of age in North-Western Tanzania: a multicentre study in a

Formatted: Indent: Left: 0", Hanging: 0.25", Line spacing: Double

Field Code Changed

Formatted: Font: (Default) Times New Roman

cascade of referral health care system. BMC Pediatr. 2019;19(1):32. Epub 20190126. doi: 10.1186/s12887-019-1411-0. PubMed PMID: 30684964; PubMed Central PMCID: PMCPMC6347777.

5. Manyahi J, Kibwana U, Mgimba E, Majigo M. Multi-drug resistant bacteria predict mortality in bloodstream infection in a tertiary setting in Tanzania. PLoS One. 2020;15(3):e0220424. Epub 20200304. doi: 10.1371/journal.pone.0220424. PubMed PMID: 32130227; PubMed Central PMCID: PMCPMC7055912.
6. Godfrey E, Majaliwa E, Assenga EN. Aetiology, antimicrobial susceptibility and outcome of children with sepsis, admitted at Muhimbili National Hospital, Dar es Salaam. Pan Afr Med J. 2022;42:167. Epub 20220701. doi: 10.11604/pamj.2022.42.167.29969. PubMed PMID: 36187027; PubMed Central PMCID: PMCPMC9482214.
7. Kortz TB, Sawe HR, Murray B, Enanoria W, Matthay MA, Reynolds T. Clinical Presentation and Outcomes among Children with Sepsis Presenting to a Public Tertiary Hospital in Tanzania. Front Pediatr. 2017;5:278. Epub 20171222. doi: 10.3389/fped.2017.00278. PubMed PMID: 29312910; PubMed Central PMCID: PMCPMC5743673.
8. Blomberg B, Manji KP, Urassa WK, Tamim BS, Mwakagile DS, Jureen R, et al. Antimicrobial resistance predicts death in Tanzanian children with bloodstream infections: a prospective cohort study. BMC Infect Dis. 2007;7:43. Epub 20070522. doi: 10.1186/1471-2334-7-43. PubMed PMID: 17519011; PubMed Central PMCID: PMCPMC1891109.
9. Blomberg B, Jureen R, Manji KP, Tamim BS, Mwakagile DS, Urassa WK, et al. High rate of fatal cases of pediatric septicemia caused by gram-negative bacteria with extended-spectrum beta-lactamases in Dar es Salaam, Tanzania. J Clin Microbiol. 2005;43(2):745-9. doi:

10.1128/jcm.43.2.745-749.2005. PubMed PMID: 15695674; PubMed Central PMCID: PMCPMC548071.

10. Pammi M, Zhong D, Johnson Y, Revell P, Versalovic J. Polymicrobial bloodstream infections in the neonatal intensive care unit are associated with increased mortality: a case-control study. BMC Infect Dis. 2014;14:390. Epub 20140714. doi: 10.1186/1471-2334-14-390. PubMed PMID: 25022748; PubMed Central PMCID: PMCPMC4226990.
11. Zhang L, Liu ZY, Xu YC, Li TS, Yang QW, Wang AX. [The microbiological and clinical analysis of bloodstream infections with identifiable sources]. Zhonghua Nei Ke Za Zhi. 2012;51(5):366-70. PubMed PMID: 22883335.
12. Lin JN, Lai CH, Chen YH, Chang LL, Lu PL, Tsai SS, et al. Characteristics and outcomes of polymicrobial bloodstream infections in the emergency department: A matched case-control study. Acad Emerg Med. 2010;17(10):1072-9. doi: 10.1111/j.1553-2712.2010.00871.x. PubMed PMID: 21040108.
13. Zheng C, Zhang S, Chen Q, Zhong L, Huang T, Zhang X, et al. Clinical characteristics and risk factors of polymicrobial *Staphylococcus aureus* bloodstream infections. Antimicrob Resist Infect Control. 2020;9(1):76. Epub 20200527. doi: 10.1186/s13756-020-00741-6. PubMed PMID: 32460851; PubMed Central PMCID: PMCPMC7254655.
14. Clinical and Laboratory Standards Institute (2018) Performance standards for antimicrobial susceptibility testing; twenty-eighth informational supplement CLSI document M100–S20. Clinical and Laboratory Standards Institute W, PA.
15. Xu J, Yuan Y, Wang B, Zhang Q, Wang J, Wang S, et al. Microbiological Analysis and Mortality Risk Factors in Patients with Polymicrobial Bloodstream Infections. Infect Drug

Formatted: Font: (Default) Times New Roman, Italic

Formatted: Font: (Default) Times New Roman

Resist. 2023;16:3917-27. Epub 20230620. doi: 10.2147/idr.S412669. PubMed PMID: 37361937; PubMed Central PMCID: PMCPMC10290474.

16. Bizzarro MJ, Dembry LM, Baltimore RS, Gallagher PG. Matched case-control analysis of polymicrobial bloodstream infection in a neonatal intensive care unit. Infect Control Hosp Epidemiol. 2008;29(10):914-20. doi: 10.1086/591323. PubMed PMID: 18808341.
17. Tsai MH, Chu SM, Hsu JF, Lien R, Huang HR, Chiang MC, et al. Polymicrobial bloodstream infection in neonates: microbiology, clinical characteristics, and risk factors. PLoS One. 2014;9(1):e83082. Epub 20140114. doi: 10.1371/journal.pone.0083082. PubMed PMID: 24454692; PubMed Central PMCID: PMCPMC3891628.
18. Nabb DL, Song S, Kluthe KE, Daubert TA, Luedtke BE, Nuxoll AS. Polymicrobial Interactions Induce Multidrug Tolerance in *Staphylococcus aureus* Through Energy Depletion. Front Microbiol. 2019;10:2803. Epub 20191205. doi: 10.3389/fmicb.2019.02803. PubMed PMID: 31866973; PubMed Central PMCID: PMCPMC6906149.
19. Little W, Black C, Smith AC. Clinical Implications of Polymicrobial Synergism Effects on Antimicrobial Susceptibility. Pathogens. 2021;10(2). Epub 20210201. doi: 10.3390/pathogens10020144. PubMed PMID: 33535562; PubMed Central PMCID: PMCPMC7912749.
20. Tay WH, Chong KK, Kline KA. Polymicrobial-Host Interactions during Infection. J Mol Biol. 2016;428(17):3355-71. Epub 20160508. doi: 10.1016/j.jmb.2016.05.006. PubMed PMID: 27170548.
21. Sutter D, Stagliano D, Braun L, Williams F, Arnold J, Ottolini M, et al. Polymicrobial bloodstream infection in pediatric patients: risk factors, microbiology, and antimicrobial

Formatted: Font: (Default) Times New Roman, Italic

Formatted: Font: (Default) Times New Roman

management. Pediatr Infect Dis J. 2008;27(5):400-5. doi: 10.1097/INF.0b013e31816591be. PubMed PMID: 18398386.

22. Song F, Zhang K, Huang J, Qian Z, Zhou H, Cai J, et al. Clinical Characteristics, Risk Factors, and Outcomes of Patients with Polymicrobial *Klebsiella pneumoniae* Bloodstream Infections. Biomed Res Int. 2021;2021:6619911. Epub 20210619. doi: 10.1155/2021/6619911. PubMed PMID: 34239928; PubMed Central PMCID: PMCPMC8235985.

23. Sancho S, Artero A, Zaragoza R, Camarena JJ, González R, Nogueira JM. Impact of nosocomial polymicrobial bloodstream infections on the outcome in critically ill patients. Eur J Clin Microbiol Infect Dis. 2012;31(8):1791-6. Epub 20111214. doi: 10.1007/s10096-011-1503-8. PubMed PMID: 22167257.

24. Goldman S, Itshaki O, Shochat T, Gafter-Gvili A, Yahav D, Rubinovitch B, et al. Risk Factors and Outcome of Polymicrobial Bacteremia: A Retrospective Cohort Study. Isr Med Assoc J. 2020;22(5):279-84. PubMed PMID: 32378818.

Formatted: Font: (Default) Times New Roman, Italic

Formatted: Font: (Default) Times New Roman

Formatted: Indent: Hanging: 0.25"

Table 1: Demographic-clinical characteristics of the participants

Variable	Monomicrobial BSIs(N=150)	Polymicrobial BSIs (N=50)	*p*-value
Age (median, IQR)	1 (1-40)	1 (1-40)	
<1month	6923	2369	
<1 year	18	6	
2–16 year	9	3	
>17 years	54	18	
Sex, Male	75 (50%)	26 (52%)	0.8
Ward, ICU	90 (60%)	31 (62%)	0.8
Comorbidity, Yes	68 (45.3%)	22 (44%)	0.87
Appropriate treatment, Yes	72 (48%)	18 (36%)	0.14

IQR- Interquartile ratio; ICU-intensive care unit

Table 2: Bacterial isolates from monomicrobial and polymicrobial bloodstream infections

Bacteria	Monomicrobial BSI N(%)	Polymicrobial BSI N(%)
K. pneumoniae	25 (16.67)	17 (17.00)
E. coli	17(11.33)	7 (7.00)
Enterobacter spp	13(8.67)	15(15.00)
*Other Enterobacterales	13 (8.67)	16 (16.00)
Acinetobacter spp	15 (15)	9 (9.00)
P. aeruginosa	14 (9.33)	8 (8.00)
#Non-Enterobacterales	4 (2.67)	6(6.00)
S. aureus	26 (17.33)	9(9.00)
CoNS	20 (13.33)	9(9.00)
Streptococcus spp	2(1.33)	3(3.00)
Candida spp	1 (0.67)	1 (1.00)
**Total**	**150**	**100**

*Other Enterobacterales: *Morganella morganii*, *Serratia mercescens*, *Providencia* spp, *Citrobacter* species #Non-Enterobacterales: *Aeromonas* spp, *Chromobacteria violaceum*; CoNS:Coagulase negative Staphylococcus

Table 3: Antimicrobial susceptibility patterns of bacteria from bloodstream infections

	Percent Resistance															
**Bacteria (n)**	**AMP**	**AMC**	**TZP**	**CRO**	**CAZ**	**FEP**	**MEM**	**AMK**	**CN**	**CIP**	**CHL**	**SXT**	**P**	**FOX**	**ERY**	**CLI**
K. pneumoniae (42)	100	80	75	72	71	76	44	39	61	69	68	59				
E. coli (24)	92	81	75	80	74	90	26	44	53	76	36	100				
Enterobacter spp (28)	100	89	60	76	81	74	79	36	70	79	69	70				
*Other Enterobacterales (29)	84	75	38	84	52	87	45	38	60	58	53	69				
Acinetobacter spp (24)			70	89	59	68	57	37	65	50	46	55				
P. aeruginosa (22)			58		39	31	24	32	58	47						
#Non-Enterobacterales (10)			63		33	44	0	22	56	22	14	67				
S. aureus (35)									60	58	28	45	100	77	80	38
CoNS (29)									62	94	16	90	100	85	89	90
**Overall**	**91**	**79**	**64**	**80**	**64**	**72**	**43**	**36**	**62**	**64**	**48**	**67**	**100**	**81**	**84**	**64**

AMK-Amikacin, AMC-Amoxycillin-clavulanic acid, AMP-Ampicillin, FOX-Cefoxitin, CAZ-Ceftazidime, CRO-Ceftriaxone, CHL-Chloramphenicol, CIP-Ciprofloxacin, CLI-Clindamycin, ERY-Erythromycin, GEN-Gentamicin, MEM-Meropenem, TZP-Tazobactam-piperacillin, P-Penicillin, SXTTrimethoprim-sulfamethoxazole. Other Enterobacterales: *Morganella morganii*, *Serratia mercescens*, *Providencia* spp, *Citrobacter* species #Non-Enterobacterales: *Aeromonas* spp, *Chromobacteria violaceum*. CoNS:Coagulase negative Staphylococcus

Table 4. Multivariable analysis assessing the association between BSIs and mortality

**Outcome**	**Monomicrobial BSIs**	**Polymicrobial BSIs**	**cOR, 95%CI, *p*-value**	**aOR, 95%CI, *p*-valu**
**All patients**	**N=150**	**N=50**		
30-days mortality (%)	30	44	1.83 (0.95-3.54), 0.07	2.05, (1.03-4.08), 0.04
In-hospital mortality (%)	31.3	50	2.19 (1.14-4.21), 0.02	2.37, (1.20-4.69), 0.01
**Neonates only**	**N=6972**	**N=23**		
30-days mortality (%)	420.0328	65.22	2.78, 1.04-7.39, 0.04	4.02, 1.32-12.23, 0.01
In-hospital mortality (%)	420.0328	73.91	4.20, 1.48-11.92, 0.007	6.18, 1.89-20.22, 0.003
**Non-neonates**	**N=81**	**N=27**		
30-days mortality (%)	19.75	25.93	1.42, 0.51-3.94, 0.50	1.55, 0.55- 4.41, 0.59
In-hospital mortality (%)	22.22	29.63	1.47, 0.55-3.91, 0.45	1.59, 0.58 - 4.36, 0.36

BSI: Bloodstream infections, cOR: crude odd ratio, aOR: adjustable odd ratio, CI: Confidence interval

Formatted Table

Formatted: Font: Bold

Table 5: Length of hospital stay post-bloodstream infections

Outcome	Monomicrobial BSIs	Polymicrobial BSIs	*p*-value
**All patients**	**N=105**	**N=28**	
LOS (median, IQR)	13 (5-24)	26 (6-37)	0.2
**Neonates only**	**N=43**	**N=8**	
LOS (median, IQR)	13 (6-32)	32 (21.5-62)	0.07

LOS: Length of hospital stay, IQR: Interquartile range, BSI: Bloodstream infections

Response to review

Review of the paper titled Polymicrobial Blood stream Infections; a risk factor for mortality at the National Hospital Tanzania; A case control study

Reviewer #1: The evidence for polymicrobial BSI is not very clear just isolation of two or more bacteria from blood culture does not substantiate the polymicrobial BSI.

Comment: Line 164 and 165 Acinetobacter and CONS, Pseudomonas and S.aureus in these combinations CONS and S.aureus could be skin contaminants in which case BSI is mono-microbial only. Therefore, selection criteria of polymicrobial BSI cases to be clearly defined in which clinical conditions or comorbidities polymicrobial BSI is a possibility only such case to be selected.
Response: Thank you for the comment. Our laboratory SoP for a blood culture states very clearly when to consider CoNS, and other possible skin contaminants as true pathogens, and we strictly adhered to the laboratory SoP in defining pathogens. S. aureus in blood culture is always considered a pathogen in our interpretation of the blood culture positive

Comment: Line 166 and 167 Acinetobacter 15% (15/150) calculation error.
Response: We appreciate for the comment, we have addressed calculation error

Comment: While mentioning the antibiotic resistance consider the antibiotics used for blood stream infections not clindamycin, cotrimoxazole and chloramphenicol for S. aureus.
Response: We appreciate for the comment. However, our results show the susceptibility pattern based on laboratory testing and not based treatment of S. aureus blood stream infections. In reporting AST results to clinician, our laboratory considers both bacteria and site of infections.

Comment: Which are the disease comorbidities were considered for statistical analysis because these disease comorbidities will play very significant role in hospital mortality and 30-day mortality in patients with BSI besides the antimicrobial resistance.
Response: Unfortunately, clinical conditions were so diverse, and a breakdown presentation was difficulties. Therefore, in our analysis we categorized either presence of comorbidity or not.

Reviewer #2: The paper made an interesting reading, dealing with polymicrobial infections Vs Monomicrobial Infections , the microbiological and clinical impact of these with respect to 30 day mortality and the extended hospital stay etc.
Comments for the same are attached , the authors are advised to kindly refer to the same and address all the points mentioned

The paper deals with the differences (Both Microbiological yield and clinical outcomes) in patients with Polymicrobial and Monomicrobial Bacteremia Infections; The Following observations are in order and must be addressed before the manuscript can be taken forward

Comment: The title of the paper does not reveal that the major component of the study is in children, adolescents, neonates, infants or adults. However, the Table No 4 & 5 refer to clinical outcomes in neonates only.
Response: Thank you for the comment. We agree that the title does not mention the major components of the participants. Our study aimed at creating awareness of the negative effects of polymicrobial bloodstreams on patient care for both laboratory personnel and clinicians. Being a lab-based retrospective, we therefore enrolled all patients with polymicrobial bloodstreams matching their counterparts by age, date of admission, and admitting wards. However, because we had a significant number of participants who were neonates, we decided to perform a sub-analysis for neonates only, as appears in Tables 4 and 5. On the other hand, we have done sub-analysis in none neonates, Therefore, we have modified the title to show the component affected.

Comment: Moreover, the Table – 1 which deals with age and sex distribution does not give a breakdown of the age groups and refers to a median age alone.
Response: Thank you for the comment. Our study design was a case control, which was matched by age; therefore, we found most participants were populated in the same age category. However, we have added age breakdown in table1

Comment: The discussion section refers to certain observations which have been compared with other studies and these deal with outcomes in neonates only.
Response: We appreciate for the comment, we have improved our citation and omitted references which do not match with our study population

Comment: In the section "Materials and Methods " "study setting and design ' the authors have stated that results are obtained in age groups from 1-86 years and of these 50 patients were selected and 150 controls. What was the age distribution taken into consideration when this selection was made. The clinical outcomes may be different in different age groups with polymicrobial bacteremia.
Response: We appreciate for the good comment, in this study we included all age groups from 0-86. Understanding the age have influence on the clinical outcome, first we adjusted for age in multivariable analysis. Then we did stratification analysis for neonate only and non-neonate. Having this analysis, we are assured we have controlled the age a possible confounder influencing our outcome of interest.

Comment: The authors have stated a definition for Polymicrobial infections in the section "Definition of terms". However, the term polymicrobial need not be restricted to 2 organisms as there are instances of blood stream infections caused by more than 2 organisms, including a yeast. How has the selection for polymicrobial infections data been made. Moreover the Results section talks of common organisms as Nos/ 100 which means that the authors have considered only 2 organisms per blood culture in a patient (total no of patients being 50).
Response: Thank you for this observation. We completely agree polymicrobial infections can be caused by two or more bacteria. Unfortunate, during the review of the laboratory data, we did not come to an instance of having more than two pathogens. Furthermore, we had one case of polymicrobial bloodstream infections involving bacteria and candida species.

Comment: The clinical outcomes for blood stream infections in conditions such as Perforation peritonitis, Carcinoma colon etc may depend on the nos of organisms causing the Polymicrobial Infection. Has this been taken into account. There is no mention anywhere in the manuscript and yeasts do not form part of the study.
Response: In our analysis we considered co-morbidities as one of the confounders, which could influence our outcome of interest, and in multivariable analysis we controlled for comorbidities, however, we found this did not influence our outcome. Furthermore, we did not document any case with perforation or peritonitis, but there was only one case of polymicrobial infections in patient with rectal carcinoma. In table 2, we have mentioned yeast being part of the study.

Comment: The authors have stated that Viridans streptococcus and Corynebacteria have been disregarded as contaminants. However, these organisms may assume huge clinical significance in certain clinical situations such as immunocompromise and malignancies in patients with BSI's. The authors have not provided a break up of clinical conditions in the 50 cases and 150controls which in itself may skew the clinical outcome data of patients.
Response: Thank you for the comment. Our laboratory SoP for a blood culture states very clearly when to consider CoNS, Viridans Streptococcus, Corynebacterium as true pathogens, and we strictly followed the lab SoP regarding these as pathogenic. Unfortunately, clinical conditions were diverse, and a breakdown presentation in a table would be difficult for a reader.

Comment: The description of Results section under "Antimicrobial Susceptibility Pattern" does not match the data outlined in Table -3.
Response: Thank you for observation, we have edited on the total number of MDR bacteria

Comment: There is no reference to certain Resistance mechanisms such as Carbapenem resistant Enterobacterales (CRE), Amp C enzymes MLSBi/c detection in S. aureus. All of these do impact treatment outcomes. How have these been factored in the selection of 50 cases and 150 controls in the study as these would most certainly affect the final analyses
Response: Thank you for the valid comment. In the selection of cases and controls, we did not consider if patients had resistant strains or not because our study hypothesized that polymicrobial bloodstream infections were associated with poor treatment outcomes, and we designed this study as a case control to answer our research question. We had thought of controlling for MDR or resistant strains, but the analysis could have been different and not for a case-control study, maybe cross-sectional.

Comment: The section on Discussion gives vague references to 30-day mortality and extended hospital stay etc without actual figures and how these may compare with similar data brought out in other studies. There are equal references to other studies in neonates without actually discussing the distribution of cases among neonates in the present study.
Response: Thank you for the comments, we have improved our reference to include reference from similar study group.

Comment: Based on the data presented and discussed the authors may have to modify the conclusion section as no definite conclusions emerge out of the discussion cited.

Response: Thank you, we have rephrased our conclusion.
