# Supplementary material for: Polymicrobial bloodstream infections a risk factor for mortality in neonates at the national hospital, Tanzania: A case-control study
Source: PLoS One. 2024 Apr 16;19(4):e0302076. doi: 10.1371/journal.pone.0302076 (PMC11020784; doi:10.1371/journal.pone.0302076)
Supplement: Attachment — Submitted filename: reviewof Plos one artice.docx [file pone.0302076.s002.docx]

**Review of the paper titled Polymicrobial Blood stream Infections ; a risk factor for mortality at the National Hospital Tanzania; A case control study**

The paper deals with the differences (Both Microbiological yield and clinical outcomes ) in patients with Polymicrobial and Monomicrobial Bacteremic Infections ; The GFollowing observations are in order and must be addressed before the manuscript can be taken forward

1. The title of the paper does not reveal that the major component of the study are in children, adolscents, neonates, infants or adults. However the Table No 4 & 5 refer to clinical outcomes in Noenates only. Moreover the Table – 1 which deals with age and sex distribution does not give a break down of the age groups and refers to a median age alone . The discussion section refers to certain observations which have been compared with other studies and these deal with outcomes in neonates only. In the section “ Materials and Methods “ “study setting and design ‘ the authors have stated that results are obtained in age groups from 1-86 years and of these 50 patients were selected and 150 controls. What was the age distribution taken into consideration when this selection was made. The clinical outcomes may be different in different age groups with polymicrobial bacteremias.
2. The authors have stated a definition for Polymicrbial infections in the section “ Definition of terms”. However the term polymicrobial need not be restricted to 2 organisms as there are instances of blood stream infections caused by more than 2 organisms, including a yeast. How has the selection for polymicrobial infections data been made .Moreover the Results section talks of common organisms as Nos/ 100 which means that the authors have considered only 2 organisms per blood culture in a patient ( total no of patients being 50 ) . The clinical outcomes for blood stream infections in conditions such as Perforation peritonitis, Carcinoma colon etc may depend on the nos of organisms causing the Polymicrobial Infection. Has this been taken into account . There is no mention anywhere in the manuscript and yeasts do not form part of the study .
3. The authors have stated that Viridans streptococcus and Corynebacteria have been disregarded as contaminants. However these organisms may assume huge clinical significance in certain clinical situations such as immunocompromise and malignancies in patients with BSI’s The authors have not provided a break up of clinical conditions in the 50 cases and 150controls which in itself may skew the clinical outcome data of patients.
4. The description of Results section under “ Antimicrobial Susceptibility Pattern” does not match the data outlined in Table -3 . There is no reference to certain Resistance mechanisms such as Carbapenem resistant Enterobacterales ( CRE), Amp C enzymes MLSBi/c detection in S. aureus . All of these do impact treatment outcomes. How have these been factored in the selection of 50 cases and 150 controls in the study as these would most certainly affect the final analyses
5. The section on Discussion gives vague references to 30 day mortality and extended hospital stay etc without actual figures and how these may compare with similar data brought out in other studies. There are equal references to other studies in neonates without actually discussing the distribution of cases among neonates in the present study.
6. Based on the data presented and discussed the authors may have to modify the conclusion section as no definite conclusions emerge out of the discussion cited.
