# Supplementary material for: Polymicrobial bloodstream infections a risk factor for mortality in neonates at the national hospital, Tanzania: A case-control study
Source: PLoS One. 2024 Apr 16;19(4):e0302076. doi: 10.1371/journal.pone.0302076 (PMC11020784; doi:10.1371/journal.pone.0302076)
Supplement: Attachment — Submitted filename: Response to reviewers comment.docx [file pone.0302076.s005.docx]

**Reviewer #3**: The authors need to mark ALL the sentences in the manuscript where they have responded to the reviewer's comments. This has not been done.
There are still some grammatical errors that need correction.

**Response**: Thank you for the comments to improve our manuscript. We have added yellow highlights in areas we have made changes in a Revised Manuscript with Track Changes. Furthermore, we have addressed some of the grammatical errors as suggested.

In addition, below are authors response from the previous reviewers

**Response to review**

**Review of the paper titled Polymicrobial Blood stream Infections; a risk factor for mortality at the National Hospital Tanzania; A case control study**

**Reviewer #1**: The evidence for polymicrobial BSI is not very clear just isolation of two or more bacteria from blood culture does not substantiate the polymicrobial BSI.

**Comment**: Line 164 and 165 Acinetobacter and CONS, Pseudomonas and S.aureus in these combinations CONS and S.aureus could be skin contaminants in which case BSI is mono-microbial only. Therefore, selection criteria of polymicrobial BSI cases to be clearly defined in which clinical conditions or comorbidities polymicrobial BSI is a possibility only such case to be selected.

**Response**: Thank you for the comment. Our laboratory SoP for a blood culture states very clearly when to consider CoNS, and other possible skin contaminants as true pathogens, and we strictly adhered to the laboratory SoP in defining pathogens. S. aureus in blood culture is always considered a pathogen in our interpretation of the blood culture positive

**Comment**: Line 166 and 167 Acinetobacter 15% (15/150) calculation error.

**Response**: We appreciate for the comment, we have addressed calculation error

**Comment**: While mentioning the antibiotic resistance consider the antibiotics used for blood stream infections not clindamycin, cotrimoxazole and chloramphenicol for S. aureus.

**Response**: We appreciate for the comment. However, our results show the susceptibility pattern based on laboratory testing and not based treatment of S. aureus blood stream infections. In reporting AST results to clinician, our laboratory considers both bacteria and site of infections.

**Comment**: Which are the disease comorbidities were considered for statistical analysis because these disease comorbidities will play very significant role in hospital mortality and 30-day mortality in patients with BSI besides the antimicrobial resistance.

**Response**: Unfortunately, clinical conditions were so diverse, and a breakdown presentation was difficulties. Therefore, in our analysis we categorized either presence of comorbidity or not.

**Reviewer #2**: The paper made an interesting reading, dealing with polymicrobial infections Vs Monomicrobial Infections , the microbiological and clinical impact of these with respect to 30 day mortality and the extended hospital stay etc.
Comments for the same are attached , the authors are advised to kindly refer to the same and address all the points mentioned

The paper deals with the differences (Both Microbiological yield and clinical outcomes) in patients with Polymicrobial and Monomicrobial Bacteremia Infections; The Following observations are in order and must be addressed before the manuscript can be taken forward

**Comment**: The title of the paper does not reveal that the major component of the study is in children, adolescents, neonates, infants or adults. However, the Table No 4 & 5 refer to clinical outcomes in neonates only.

**Response**: Thank you for the comment. We agree that the title does not mention the major components of the participants. Our study aimed at creating awareness of the negative effects of polymicrobial bloodstreams on patient care for both laboratory personnel and clinicians. Being a lab-based retrospective, we therefore enrolled all patients with polymicrobial bloodstreams matching their counterparts by age, date of admission, and admitting wards. However, because we had a significant number of participants who were neonates, we decided to perform a sub-analysis for neonates only, as appears in Tables 4 and 5. On the other hand, we have done sub-analysis in none neonates, Therefore, we have modified the title to show the component affected.

**Comment**: Moreover, the Table – 1 which deals with age and sex distribution does not give a breakdown of the age groups and refers to a median age alone.

**Response**: Thank you for the comment. Our study design was a case control, which was matched by age; therefore, we found most participants were populated in the same age category. However, we have added age breakdown in table1

**Comment**: The discussion section refers to certain observations which have been compared with other studies and these deal with outcomes in neonates only.

**Response**: We appreciate for the comment, we have improved our citation and omitted references which do not match with our study population

**Comment**: In the section “Materials and Methods “ “study setting and design ‘ the authors have stated that results are obtained in age groups from 1-86 years and of these 50 patients were selected and 150 controls. What was the age distribution taken into consideration when this selection was made. The clinical outcomes may be different in different age groups with polymicrobial bacteremia.

**Response**: We appreciate for the good comment, in this study we included all age groups from 0-86. Understanding the age have influence on the clinical outcome, first we adjusted for age in multivariable analysis. Then we did stratification analysis for neonate only and non-neonate. Having this analysis, we are assured we have controlled the age a possible confounder influencing our outcome of interest.

**Comment**: The authors have stated a definition for Polymicrobial infections in the section “Definition of terms”. However, the term polymicrobial need not be restricted to 2 organisms as there are instances of blood stream infections caused by more than 2 organisms, including a yeast. How has the selection for polymicrobial infections data been made. Moreover the Results section talks of common organisms as Nos/ 100 which means that the authors have considered only 2 organisms per blood culture in a patient (total no of patients being 50).

**Response:** Thank you for this observation. We completely agree polymicrobial infections can be caused by two or more bacteria. Unfortunate, during the review of the laboratory data, we did not come to an instance of having more than two pathogens. Furthermore, we had one case of polymicrobial bloodstream infections involving bacteria and candida species.

**Comment**: The clinical outcomes for blood stream infections in conditions such as Perforation peritonitis, Carcinoma colon etc may depend on the nos of organisms causing the Polymicrobial Infection. Has this been taken into account. There is no mention anywhere in the manuscript and yeasts do not form part of the study.

**Response**: In our analysis we considered co-morbidities as one of the confounders, which could influence our outcome of interest, and in multivariable analysis we controlled for comorbidities, however, we found this did not influence our outcome. Furthermore, we did not document any case with perforation or peritonitis, but there was only one case of polymicrobial infections in patient with rectal carcinoma. In table 2, we have mentioned yeast being part of the study.

**Comment**: The authors have stated that Viridans streptococcus and Corynebacteria have been disregarded as contaminants. However, these organisms may assume huge clinical significance in certain clinical situations such as immunocompromise and malignancies in patients with BSI’s. The authors have not provided a break up of clinical conditions in the 50 cases and 150controls which in itself may skew the clinical outcome data of patients.

**Response**: Thank you for the comment. Our laboratory SoP for a blood culture states very clearly when to consider CoNS, Viridans Streptococcus, Corynebacterium as true pathogens, and we strictly followed the lab SoP regarding these as pathogenic. Unfortunately, clinical conditions were diverse, and a breakdown presentation in a table would be difficult for a reader.

**Comment:** The description of Results section under “Antimicrobial Susceptibility Pattern” does not match the data outlined in Table -3.

**Response**: Thank you for observation, we have edited on the total number of MDR bacteria

**Comment:** There is no reference to certain Resistance mechanisms such as Carbapenem resistant Enterobacterales (CRE), Amp C enzymes MLSBi/c detection in S. aureus. All of these do impact treatment outcomes. How have these been factored in the selection of 50 cases and 150 controls in the study as these would most certainly affect the final analyses

**Response**: Thank you for the valid comment. In the selection of cases and controls, we did not consider if patients had resistant strains or not because our study hypothesized that polymicrobial bloodstream infections were associated with poor treatment outcomes, and we designed this study as a case control to answer our research question. We had thought of controlling for MDR or resistant strains, but the analysis could have been different and not for a case-control study, maybe cross-sectional.

**Comment**: The section on Discussion gives vague references to 30-day mortality and extended hospital stay etc without actual figures and how these may compare with similar data brought out in other studies. There are equal references to other studies in neonates without actually discussing the distribution of cases among neonates in the present study.

**Response**: Thank you for the comments, we have improved our reference to include reference from similar study group.

**Comment**: Based on the data presented and discussed the authors may have to modify the conclusion section as no definite conclusions emerge out of the discussion cited.

**Response**: Thank you, we have rephrased our conclusion.
